# Microbial Community and *in situ* Bioremediation of Groundwater by Nitrate Removal in the Zone of a Radioactive Waste Surface Repository

**DOI:** 10.3389/fmicb.2018.01985

**Published:** 2018-08-23

**Authors:** Alexey V. Safonov, Tamara L. Babich, Diyana S. Sokolova, Denis S. Grouzdev, Tatiyana P. Tourova, Andrey B. Poltaraus, Elena V. Zakharova, Alexander Y. Merkel, Alexander P. Novikov, Tamara N. Nazina

**Affiliations:** ^1^Frumkin Institute of Physical Chemistry and Electrochemistry, Russian Academy of Sciences, Moscow, Russia; ^2^V.I. Vernadsky Institute of Geochemistry and Analytical Chemistry of Russian Academy of Sciences, Moscow, Russia; ^3^Winogradsky Institute of Microbiology, Research Center of Biotechnology, Russian Academy of Sciences, Moscow, Russia; ^4^Institute of Bioengineering, Research Center of Biotechnology of the Russian Academy of Sciences, Moscow, Russia; ^5^Engelhardt Institute of Molecular Biology, Russian Academy of Sciences, Moscow, Russia

**Keywords:** microbial ecology, surface radioactive waste repository, high-throughput sequencing, 16S rRNA gene, denitrifying bacteria, *nirS* and *nirK* genes, nitrate removal, *in situ* groundwater bioremediation

## Abstract

The goal of the present work was to investigate the physicochemical and radiochemical conditions and the composition of the microbial community in the groundwater of a suspended surface repository for radioactive waste (Russia) and to determine the possibility of *in situ* groundwater bioremediation by removal of nitrate ions. Groundwater in the repository area (10-m depth) had elevated concentrations of strontium, tritium, nitrate, sulfate, and bicarbonate ions. High-throughput sequencing of the V3–V4/V4 region of the 16S rRNA gene revealed the presence of members of the phyla *Proteobacteria* (genera *Acidovorax, Simplicispira, Thermomonas, Thiobacillus, Pseudomonas, Brevundimonas*, and uncultured *Oxalobacteraceae*), *Firmicutes* (genera *Bacillus* and *Paenibacillus*), and *Actinobacteria* (*Candidatus* Planktophila, *Gaiella*). Canonical correspondence analysis suggested that major contaminant – nitrate, uranium, and sulfate shaped the composition of groundwater microbial community. Groundwater samples contained culturable aerobic organotrophic, as well as anaerobic fermenting, iron-reducing, and denitrifying bacteria. Pure cultures of 33 bacterial strains belonging to 15 genera were isolated. Members of the genera *Pseudomonas, Rhizobium, Cupriavidus, Shewanella, Ensifer*, and *Thermomonas* reduced nitrate to nitrite and/or dinitrogen. Application of specific primers revealed the *nirS* and *nirK* genes encoding nitrite reductases in bacteria of the genera *Pseudomonas, Rhizobium*, and *Ensifer*. Nitrate reduction by pure bacterial cultures resulted in decreased ambient Eh. Among the organic substrates tested, sodium acetate and milk whey were the best for stimulation of denitrification by the microcosms with groundwater microorganisms. Injection of these substrates into the subterranean horizon (single-well push-pull test) resulted in temporary removal of nitrate ions in the area of the suspended radioactive waste repository and confirmed the possibility for *in situ* application of this method for bioremediation.

## Introduction

The methods used in Russia, United States, and other countries for disposal of radioactive waste (RAW) during the 20th century resulted in numerous repositories which represent a certain environmental danger (Rybal’chenko et al., 1998; Wall and Krumholz, 2006). Liquid RAW, responsible for most of the low- and medium- activity waste represent the greatest environmental risk; over 90% of these are technological and deactivation waste. Apart from radioactive elements, liquid RAW contains inorganic and organic salts (sodium, calcium, iron, and ammonium acetate, sulfate, chloride, phosphate, oxalate, and bicarbonate), with total salinity reaching up to 300–500 g/dm^3^ (Rybal’chenko et al., 1998). Since nitric acid is used in uranium recovery and processing, nitrate is the main component of liquid waste, with its concentrations varying from 1 to 350 g/dm^3^. Non-radioactive waste components migrate with groundwater, covering significant distances and creating the risk of contamination of surrounding areas.

All RAW components migrating into the environment may interact with microorganisms, which are capable to affect their state and degree of migration. Ecological investigation of microorganisms in RAW-contaminated soils, salt marshes, and subsurface and deep horizons was carried out (Wall and Krumholz, 2006; Simonoff et al., 2007; Newsome et al., 2014). The microorganisms of nitrate- and radionuclide-contaminated subterranean water-bearing strata and sediments have been studied in detail (Anderson et al., 2003; Elias et al., 2003; Nevin et al., 2003; Yan et al., 2003; North et al., 2004; Fields et al., 2005; Vrionis et al., 2005; Hwang et al., 2009). Denitrifying bacteria of the genera *Afipia, Hyphomicrobium, Rhodanobacter, Intrasporangium*, and *Bacillus* were isolated from Oak Ridge sediments, Oak Ridge, TN, United States (The Field Research Center; OR-FRC^1^) (Green et al., 2010). Analysis of the *nir*K and *nir*S nitrite reductase functional genes revealed predominance of *Rhodanobacter* spp. in these sediments (Green et al., 2012).

Denitrification is carried out by phylogenetically diverse groups of autotrophic and heterotrophic microorganisms, depending on the presence of hydrogen or organic compounds. Denitrifying bacteria obtain energy by reducing nitrate to dinitrogen or other gaseous nitrogen compounds in the chain of reactions, which includes nitrite (${\mathrm{NO}}_{2}^{-}$), nitric oxide (NO), and nitrous oxide (N_2_O) as intermediates (Zumft, 1997):

$${\mathrm{NO}}_{3}^{-}\;\to \;{\mathrm{NO}}_{2}^{-}\;\to \;\mathrm{NO}\;\to \;N_{2}O\;\to \;N_{2}$$

The products of nitrate reduction are known to promote the oxidation of reduced metals and radionuclides, enhancing their migration (DiChristina, 1992). No uranium reduction was found to occur in uranium- and nitrate-contaminated sediments prior to nitrate removal (Finneran et al., 2002; Senko et al., 2002, 2005a,b; Elias et al., 2003; Wu et al., 2006, 2010). Thus denitrification, which is energetically a more profitable process than metal or radionuclide reduction, should be the first stage of bioremediation of the habitats contaminated with radionuclides and nitrates.

Experiments were carried out on nitrate removal from the basins and water-bearing horizons contaminated with the waste of nuclear fuel industry (Hanford and Oak Ridge sites, Oak Ridge, TN, United States, etc.) (Robertson and Anderson, 1999; Istok et al., 2004; Fruchter et al., 2006). Pilot trials of the method for *in situ* removal of uranium and nitrate (Oak Ridge, TN, United States) revealed reduction of redox-sensitive metals to low-valency forms with low mobility after nitrate reduction to dinitrogen (Wu et al., 2006). In laboratory and field experiments on nitrate removal, soluble (sugar, molasses, sodium lactate, and ethanol) and insoluble substrates (vegetable oil, wood, and mulch) were used as electron donors for denitrifying bacteria (Kim et al., 2005; Lloyd et al., 2005; Rocca et al., 2007; Akob et al., 2008; McMillan et al., 2012). The concept of a permeable reactive barrier was proposed as the main approach to *in situ* groundwater treatment (Powell et al., 1998; Benner et al., 1999). The stimulating substrates are injected into the water-bearing horizon, and the plume of contaminated groundwater passing through this zone is decontaminated *in situ* by the microbial community. Duration of groundwater treatment in the zone of the biological barrier is determined by hydrodynamic conditions and the amount of the substrate introduced (once or repeatedly).

In Russia, microorganisms of groundwater of deep disposal sites for liquid RAW located at the depth of 160–280 m at the Mining and Chemical Combine and Siberian Chemical Combine were studied (Nazina et al., 2004, 2010). Information on the microorganisms of contaminated groundwater located at the depth of 10–40 m is scarce. The concentrations of nitrate ions in groundwater from the 10-m depth in the areas of surface repositories for liquid RAW may exceed the maximal permissible concentrations (MPCs) by two orders of magnitude. No data on biodiversity of the microbial community in groundwater in the area of a surface RAW repository were available. Investigation of the composition and activity of microbial populations is essential for development of biotechnologies for *in situ* bioremediation of contaminated ecosystems. The goal of the present work was to investigate the physicochemical and radiochemical parameters and microbial biodiversity in nitrate- and radionuclide-contaminated groundwater collected in the area of a suspended surface repository for RAW (Russia), to determine the optimal substrates for denitrifying microcosms, and to test the biotechnology for *in situ* nitrate removal from groundwater.

## Materials and Methods

### Site and Sample Description

**FIGURE 1:**
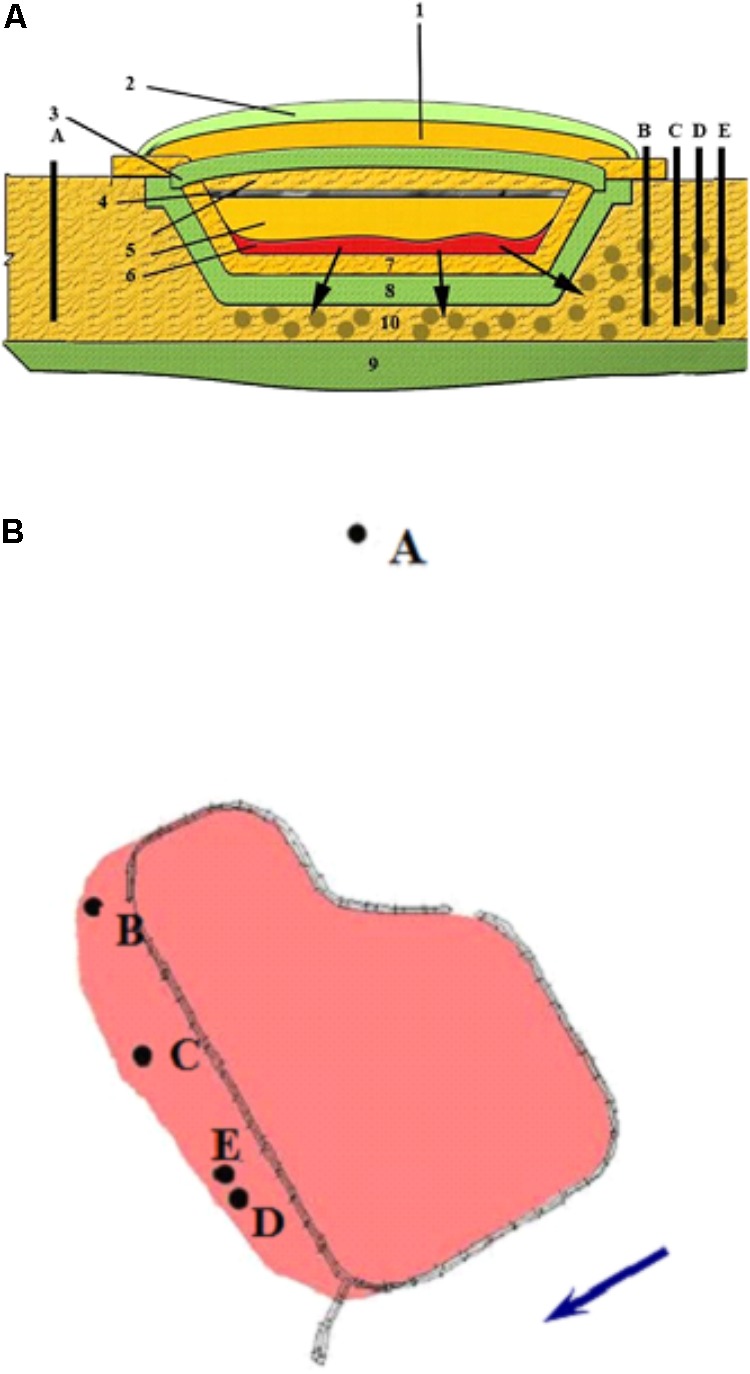
Schematic representation of the suspended surface repository for liquid radioactive waste **(A)** and location of the observation wells **(B)** outside the zone of waste spreading (well A) and in the contamination zone (wells B, C, D, and E). The direction of groundwater movement is indicated by an arrow. (1) sandy protective layer; (2) plant layer; (3) upper clay screen; (4) crushed stone; (5) sandy soil; (6) pulp; (7) gravel-sand rammed layer; (8) clay screen; (9) waterproof layer; (10) zone of waste spread.

Groundwater samples were collected from the upper water-bearing horizon (10 m depth) from five observation wells in the area of a suspended surface repository for RAW (Russia) (**Figures 1A,B**). The repository (basin) was constructed in 1964 for collection and temporary storage of RAW prior to underground burial. The repository is a specially constructed hydraulic structure, with a clay screen at the bottom and slopes covered with a protective sand layer. The repository was suspended in 2012. The natural flow of groundwater is in the southern and southwestern direction at the rate of 3–5 m per year. The temperature of the water-bearing horizon is 10°C, with annual temperature differences not exceeding 0.14°C. The groundwater pH is close to neutral (6.2 to 7.5). Eh varies from -20 to +150 mV, with the average value of +50 to +70 mV, so the redox conditions of this horizon may be characterized as microaerobic to anoxic. Contaminated groundwater was collected into a sterile 50-L bottle, from which the samples for microbiological and physicochemical analyses were subsequently taken.

### Enumeration and Isolation of Microorganisms

Abundance of the major metabolic groups of microorganisms (aerobic organotrophic and anaerobic denitrifying, fermenting, iron-reducing, sulfur-reducing, and methanogenic prokaryotes) in groundwater samples was determined by inoculating 10-fold dilutions of groundwater samples (in triplicate) into liquid nutrient media. Microbial numbers were calculated according to the most probable number technique using McCready tables (Koch, 1994). Aerobic organotrophs were enumerated in liquid medium (TEG) containing (g⋅l^-1^): bacto tryptone, 5.0; yeast extract, 2.5; and glucose, 1.0; pH 7.0. Fermenting bacteria were enumerated in the medium with peptone (4 g l^-1^) and glucose (10 g l^-1^) (Postgate, 1984); bacterial growth was assessed by microscopy and H_2_ production. Sulfate-reducing bacteria were detected by sulfide increase in the dilution series in Postgate B medium (Postgate, 1984) with sodium lactate (4 g l^-1^) and 200 mg l^-1^Na_2_S 9H_2_O as a reducing agent. Denitrifying bacteria were assessed by nitrogen production in Adkins medium (Adkins et al., 1992) supplemented with sodium acetate (2 g l^-1^) and sodium nitrate (0.85 g l^-1^). Methanogens were detected by CH_4_ production in the medium (Zeikus et al., 1975) with sodium acetate (2 g l^-1^) or H_2_/CO_2_ (80:20% vol/vol), trace elements (Pfennig and Lippert, 1966), and yeast extract (1 g l^-1^). The media for aerobic and anaerobic bacteria were prepared in Hungate tubes, with air or argon, respectively, as the gas phase (except for the medium for methanogens with H_2_/CO_2_). All tubes were incubated at room temperature until the onset of growth, but not longer than 30 days; the cultures were then examined under an Olympus phase contrast microscope (Japan), and specific microbial metabolites were analyzed.

Enrichment cultures of denitrifying bacteria were obtained in liquid medium containing the following (g⋅l^-1^): NaCl, 0.8; KCl, 0.1; NaNO_3_, 4.0; Na_2_SO_4_, 0.1; MgSO_4_, 0.1; KH_2_PO_4_, 0.6; and K_2_HPO_4_, 1.0. Organic substrates used were sodium acetate (2.8 g/L), methanol (0.2% vol/vol), ethanol (0.2% vol/vol), ammonium oxalate (2.5 g l^-1^), sodium lactate (2.5 g l^-1^), glucose (5.0 g l^-1^), sucrose (5.0 g l^-1^), milk whey (2.5% vol/vol), kefir whey (2.5% vol/vol), or yeast extract (2 g l^-1^). Milk and kefir wheys were obtained by heating sour milk and kefir on a water bath and decanting the liquid, which was then used in the experiments. The wheys had pH 4.0 and 4.75, and prior to adding then to groundwater samples they were neutralized to pH 7 with 0.1N NaOH. Milk whey contains 6.5% dry matter, including 4.5% lactose, 0.9% protein, 0.2% fat, and 0.6% mineral salts, and is a full-fledged substrate for microorganisms. The cultures were incubated at room temperature and at 10°C (under conditions similar to those occurring in the subterranean horizon). All experiments were carried out in triplicate.

Pure cultures of aerobic organotrophs and denitrifying bacteria were isolated under aerobic conditions in TEG medium by sequential transfers of well-isolated colonies from solid to liquid medium. Ability of the strains to carry out denitrification was then tested under anoxic conditions in Adkins medium with nitrate and organic substrates (acetate, lactate, or milk whey).

### Analytical Methods

Biomass growth in liquid media was assessed as OD_660_ on an Ultrospec 2100 pro spectrophotometer (Amersham Biosciences, United Kingdom). Hydrogen, nitrogen, and methane were analyzed by gas chromatography as described previously (Bonch-Osmolovskaya et al., 2003). Sulfide was analyzed colorimetrically with *N,N-*dimethyl-*p*-phenylenediamine according to the Pachmayr method in the modification of Trüper and Schlegel (1964). Concentrations of nitrate anions, pH, and Eh were determined using an Anion ionometer (Novosibirsk, Russia) with relevant electrodes. Nitrite anion was measured colorimetrically with the Griess reagent on an Ultrospec 2100 pro spectrophotometer (Amersham Biosciences, United Kingdom) at 540 nm. Chemical analysis of total organic carbon (TOC) in groundwater samples was performed by wet incineration by Tyurin according to GOST (Russian State Standard) 26213-91, following the standard procedures by the Russian Federation Gosstandart.

Minor components of the groundwater samples were analyzed on an Elan-6100 mass spectrometer with inductively coupled plasma (Perkin Elmer, United States) and an Optima-4300 DV atomic emission spectrometer with inductively coupled plasma (Perkin Elmer, United States). Anions in the samples were determined using a KAPEL^®^-104T capillary electrophoresis system (Lumeks, Russia). Cesium concentration was measured on a laboratory digital gamma-spectral complex (ORTEC, United States); other radionuclides were analyzed on a Tri-Carb-3180 TR/SL scintillation radiometer (Perkin-Elmer, United States). Radiometric measurements were also carried out on a low-background setup consisting of a spectrometer with detectors of ultrapure Ge (Canberra, Ind., Inc.) and a TriCarb -2700 liquid scintillation spectrometer (Packard, Ind., United States). All chemical and radiochemical analyses were carried out in three and six replicates, respectively.

### Identification of Pure Cultures

Pure bacterial cultures were identified by analysis of their 16S rRNA gene sequences. DNA isolation, amplification, and sequencing of the 16S rRNA genes were carried out as described previously (Nazina et al., 2006). The full-sized 16S rRNA gene was amplified using the 8f–1492r primers and was used as a template for DNA sequencing with the primers 8f, 519r, 519f, and 1492r. Preliminary analysis of the 16S rRNA gene sequences was carried out using the Ribosomal Database Project (RDP^2^) data and software. The sequences were edited using BioEdit^3^ and aligned with those of closely related bacterial species using CLUSTALW v. 1.75.

### Detection of the *nirK* and *nirS* Genes in the Strains

DNA isolated from pure cultures was used for detection of nitrite reductase genes (*nirK* and *nirS*). Polymerase chain reaction (PCR) was carried out using the primers nirK1F/nirK5R and nirS1F/nirS6R (Braker et al., 1998), nirK517F/nirK1055R (Michotey et al., 2000; Chen et al., 2010), and nirScd3aF/nirSR3cd (Michotey et al., 2000; Throbäck et al., 2004) (**Supplementary Table S1**). PCR was carried out on an iCycler (Bio-Rad Laboratories, United States) in 10 μL of the mixture containing 1× Taq buffer [10 mM Tris-HCl pH 8.3, 50 mM KCl], 2 mM MgCl_2_, 200 μM deoxyribonucleotide triphosphates, 5 pmol each of the 5′ and 3′ terminal primers, 1 AU Taq DNA polymerase (Perkin-Elmer, United States), and template DNA. The fragment length was determined in 1.0% agarose gel with ethidium bromide. M24 (SibEnzime, Russia) was used as a DNA length marker.

### High-Throughput Sequencing of the 16S rRNA Gene Fragments of Groundwater Microorganisms

#### DNA Isolation, Amplification, and Sequencing of the 16S rRNA Genes

Groundwater samples were fixed with ethanol (1 : 1 vol/vol) and filtered through 0.22-μm membranes (Millipore, United States). The cells were washed off the filters with the lysing solution containing 0.15 M NaCl and 0.1 M Na_2_EDTA [pH 8.0] and were used for DNA isolation according to the standard procedure (Maniatis et al., 1984).

To obtain the library of the 16S rRNA gene fragments, the V3–V4 hypervariable region of the gene was amplified, and the libraries were created using double barcoding, as described previously (Fadrosh et al., 2014). For samples obtained in 2016, the sense sites of the primers were taken according to the Pro341F–Pro805R primer pair (Takahashi et al., 2014). For samples obtained in 2017, total genomic DNA was amplified using the 515f/806r primer set that amplifies the V4 region of the 16S rRNA gene (Bergmann et al., 2011). The 16S rRNA gene fragments were amplified using 5× Taq Red buffer and HS Taq polymerase (Evrogen, Russia). The reaction mixture contained 5 μL of each primer (6 μM concentration), 5 μL DNA solutions, and 15 μL PCR mix (1 U polymerase, 0.2 mM of each dNTP, and 2.5 mM Mg^2+^). Each DNA sample was amplified in three replicates, which were then pooled together and purified by electrophoresis in 2% agarose gel and the Cleanup Standard gel extraction kit (Evrogen, Russia). Sequencing was carried out using the MiSeq platform (Illumina, United States) and the MiSeq Reagent Kit v3 (600 cycles) (Illumina, United States) according to the manufacturer’s recommendations. From DNA isolated from each groundwater sample collected in 2016 and in 2017, one and two libraries, respectively, were constructed. From groundwater sampled in 2016 from wells B, E, and D were constructed the libraries gwB-16, gwE-16, and gwD-16 [groundwater (gw)], respectively. From groundwater samples collected in 2017 from wells A, B, E, and D were constructed the libraries gwA-17a and gwA-17b, gwB-17a and gwB-17b, gwE-17a and gwE-17b, gwD-17a and gwD-17b, respectively.

### Bioinformatic Analysis

The 16S rRNA gene fragments (reads) were trimmed using trimmomatic-0.36 according to the SLIDINGWINDOW:4:15 algorithm (Bolger et al., 2014); right and left reads were combined using SeqPrep^4^. Bacterial and archaeal 16S rRNA gene fragments were processed using a combination of QIIME (Caporaso et al., 2010) and USEARCH (Edgar, 2010) pipelines. After demultiplexing and quality control, sequences were dereplicated and singletons were removed. Sequences were *de novo* clustered into OTUs as having ≥ 98% sequence identity using UPARSE (Edgar, 2013). Final OTU sequences were aligned using MUSCLE (Edgar, 2004) after removal of any chimeric sequences and an OTU table was generated with read frequencies. Primary taxonomic assignment was accomplished using the UCLUST algorithm (Edgar, 2004) and greengenes database (DeSantis et al., 2006). Comparison with the GenBank sequences was carried out using BLAST^5^. Phylogenetic trees were constructed using the neighbor-joining algorithm implemented in the MEGA 6.0 software package (Tamura et al., 2013).

### Statistical Analysis

Library coverage was calculated as C = 1 – (n/N), where n is the number of OTUs represented by single sequences and N is the total number of analyzed sequences (Good, 1953). Alpha diversity metrics and rarefaction curves were calculated using the QIIME (Caporaso et al., 2010). Diversity indices of the 16S rRNA gene fragments of *Bacteria* in libraries of 2017 presented in **Table 2** are the average results for two libraries for each groundwater sample. ClustVis was employed to generate the PCA plot and heat map of most abundant genera (Metsalu and Vilo, 2015). Canonical correspondence analysis (CCA) was performed to measure geochemical properties that have the most significant influence on microbial communities. CCA was conducted with package vegan version 2.5-2 (Oksanen et al., 2012).

### Nucleotide Sequence Accession Numbers

The 16S rRNA gene sequences of bacterial isolates were deposited to GenBank under Accession Nos. MG051295–MG051324; the libraries of the 16S rRNA gene fragments of the microorganisms from groundwater and enrichment cultures obtained by high-throughput sequencing were deposited to NCBI SRA under Accession No. SRP119496.

## Results and Discussion

### Physicochemical and Microbiological Characteristics of Groundwater

Groundwater samples were collected from the observation wells located within the zone of contamination with solutions from the underground RAW repository (wells B, C, D, and E) and outside this zone (well A) (**Figure 1**). Chemical and radiochemical analysis of the samples from wells B–E indicated the effect of the RAW repository on the environment (**Table 1**). This was evident from elevated concentrations of nitrate (650–4239 mg l^-1^), sulfate (16–152 mg l^-1^), and bicarbonate ions (up to 436 mg l^-1^) compared to the values for well A samples. Nitrate was the major contaminant and its concentration exceeded significantly the MPC value (45 mg l^-1^ for groundwater in Russia), which necessitated the procedures for its removal from groundwater. Iron concentrations varied within a narrow range from <0.02 to 9.93 mg l^-1^. Strontium and tritium were detected among the groundwater radionuclides. The concentrations of uranium, ^137^Cs, and ^60^Co in the studied groundwater samples were low. The total concentration of organic compounds in groundwater samples measured when the biotechnological treatment was not applied varied from 2.05 to 14.0 mg l^-1^, which indicated the oligotrophic nature of groundwater in the area of RAW repository.

**Table 1:** Chemical and radiochemical composition of groundwater samples from observation wells in the area of a suspended RAW repository.

Parameters	Standard error	Well											
		
		A		B			C			D		E	
						
		Sampling date											
		
		2013.10	2017.07	2013.10	2016.06	2017.07	2013.10	2016.06	2017.07	2016.06	2017.07	2016.06	2017.07
Total salinity, mg⋅l^-1^		127	109	7000		2300	5070		3952		894		1845
TOC, mg⋅l^-1^	±0.05	9.93			2.19			12.0		2.05		4.15	
pH	±0.1	7.06	6.41	5.91	6.06	6.33	6.20	6.56	6.58	6.63	6.83	6.73	6.75
Concentration, mg⋅l^-1^													
Fe_total_	±0.004	9.93	2.38	0.10	4.33	0.41	<0.02	0.17	0.25	0.24	0.08	0.09	0.13
Na^+^	±0.02	0.49	3.41	570.0	245.0	233.0	760.0	639.0	604.0	107.6	36.2	506.0	324.0
K^+^	±0.02	4.06	0.59	10.40	6.01	5.70	2.97	4.89	3.09	5.14	3.97	4.79	3.30
Ca^2+^	±0.09	<0.50	15.4	772.0	305.0	205.0	375.0	308.6	316.6	130.0	126.3	128.6	85.9
Mg^2+^	±0.09	17.5	2.8	145.8	58.7	56.2	63.4	65.6	63.2	38.1	34.0	30.0	20.7
${\mathrm{NH}}_{4}^{-}$	±0.09	3.64	<0.5	9.35	1.78	<0.5	9.25	6.20	7.64	1.90	0.35	1.40	<0.5
${\mathrm{HCO}}_{3}^{-}$	±0.09	<0.5	0.77	4239	1500	1172	3280	2680	2517	650	251	1426	785
${\mathrm{SO}}_{4}^{2}$	±0.02	<0.2	0.84	133.0	152.0	67.3	46.0	79.3	72.4	16.9	24.6	40.5	7.6
Cl^-^	±0.004	1.79	2.26	7.01	1.40	1.65	6.68	4.42	4.52	2.74	7.10	4.20	3.65
${\mathrm{HCO}}_{3}^{-}$	±0.001	<0.1	67.1	170.9	241.0	295.9	140.3	357.0	331.0	216.6	357.0	436.2	439.2
CO_2_	±0.23	79.3	19.4	134.9	100.8	109.1	64.0	67.7	60.8	72.9	43.9	60.2	63.3
Radioactivity, Bq⋅l^-1^													
^137^Cs	±0.001	0.05	0.05	0.41	<0.01	<0.01	0.02	<0.01	<0.01	<0.01	<0.01	<0.01	<0.01
^90^Sr	±0.001	0.04	<0.01	72.0	1.2	<0.01	<0.01	<0.01	<0.01	0.05	0.05	2.00	0.56
T	±0.02	1.3	1.3	6579	1364	1296	4543	993	2070	411	535	396	197
U(sum)	±0.001	<0.01	<0.01	<0.01	0.07	<0.01	0.01	0.72	0.05	0.39	0.04	1.78	0.05


Groundwater samples from wells A–D were found to contain culturable aerobic organotrophic (10^5^–10^6^ cells⋅ml^-1^) bacteria, as well as anaerobic fermenting (10–10^6^ cells⋅ml^-1^), iron-reducing (≥10^3^ cells⋅ml^-1^), and denitrifying bacteria (0– ≥10^4^ cells⋅ml^-1^) (**Supplementary Figure S1**). Due to oxidative conditions in the studied horizon, sulfate-reducing and methanogenic prokaryotes were almost absent. High abundance of denitrifying bacteria in natural groundwater and in contaminated groundwater in the area of the suspended RAW repository indicates the potential possibility for denitrification in this environment.

### Denitrifying Microcosms From Groundwater

In order to determine organic substrates stimulating the growth of subterranean denitrifying communities, groundwater samples from wells A, B, and C with different initial nitrate concentrations were supplemented with alcohols, organic acids, sugars, milk, and kefir whey, etc. The sample from well A, which initially did not contain nitrates, was also supplemented with sodium nitrate (1000 mg ${\mathrm{NO}}_{3}^{-}$⋅l^-1^). Denitrifying microcosms obtained using substrate-enriched groundwater reduced nitrate ions forming nitrite ions as intermediate products (**Supplementary Table S2**). At longer incubation periods, the most efficient nitrate removal with N_2_ formation was observed for the microcosms supplemented with milk whey, sucrose, and acetate.

Enrichment cultures obtained by transfers of the material from denitrifying microcosms from wells A, B, and C reduced nitrate ions in the medium with milk whey within the temperature range from 7 to 37–40°C, with the maximal nitrate removal at 10–25°C. Enrichment cultures with acetate, sucrose, or milk whey as substrates showed the best growth at pH form 6.0 to 9.0. The denitrifying enrichment enrB obtained using the material from well B grew at 13°C, i.e., at the temperature close to the ambient one in the well, on milk whey in the presence of elevated nitrate concentrations (4–10 g l^-1^) (data not shown).

### Groundwater Microbial Community

High-throughput sequencing of the 16S rRNA gene fragments was used for analysis of microbial communities in groundwater samples collected in 2016 and in 2017. The libraries contained from ∼25000 to 29798 sequences (reads). The Silva online resource was used to find unidentified sequences, which were removed from analysis. A total of 246734 valid reads originating from seven groundwater samples were classified from the phylum to the genus level according to the QIIME classifier. The diversity indices were determined for 11 libraries. In all obtained libraries Good’s coverage value was in a range of 98–99% (**Table 2**). The Chao 1 diversity indexes and rarefaction curves (**Supplementary Figure S2**) did not reach the maximal values. Diversity indices of the 16S rRNA genes were high for all studied libraries from contaminated groundwater. On the contrary, the Berger–Parker dominance index was higher for the gwE-17a, gwE-17b, and gwB-16 libraries then for other libraries, indicating the relatively low Evenness of these libraries, in which 1–3 phylotypes predominated.

**Table 2:** Diversity indices of the 16S rRNA gene fragments of members of the *Bacteria* domain in the library created based on microbial DNA from nitrate- and radionuclide-contaminated groundwater.

Parameters	*Bacteria* sequences in the library										
	
	Well A		Well B			Well D			Well E		
				
Library	gwA-17a	gwA-17b	gwB-16	gwB-17a	gwB-17b	gwD-16	gwD-17a	gwD-17b	gwE-16	gwE-17a	gwE-17b
Number of sequences in the library	19060	11092	25000	35340	18798	24999	20291	29798	25000	29189	12351
Number of OTUs	610	495	192	738	608	334	467	417	393	220	154
Good coverage (%)	99	98	99	99	98	99	99	99	99	99	99
Chao1	896 ± 307	830 ± 407	337 ± 187	920 ± 84	840 ± 155	395 ± 58	571 ± 108	575 ± 206	468 ± 76	451 ± 176	353 ± 128
Shannon-Weaver diversity index (H)	7.01	6.72	2.93	5.78	5.95	4.98	5.43	5.44	5.36	1.17	1.03
Inverse Simpson’s index (1/S)	37.45	30.96	4.07	13.51	14.39	12.00	17.76	17.70	14.99	1.32	1.26
Evenness (H/Hmax)	0.76	0.75	0.39	0.63	0.62	0.59	0.63	0.62	0.62	0.15	0.14
Berger–Parker dominance index (D)	0.10	0.12	0.39	0.22	0.22	0.21	0.157	0.157	0.193	0.87	0.89


Bacterial 16S rRNA genes predominated in all 11 libraries. The share of archaea did not exceed 0.7%, and their OTUs (phylotypes) were represented mainly by scarce sequences of uncultured *Euryarchaeota* and *Thaumarchaeota*. Only bacterial 16S rRNA gene sequences were used in the taxonomic and statistical analysis of the libraries.

**FIGURE 2:**
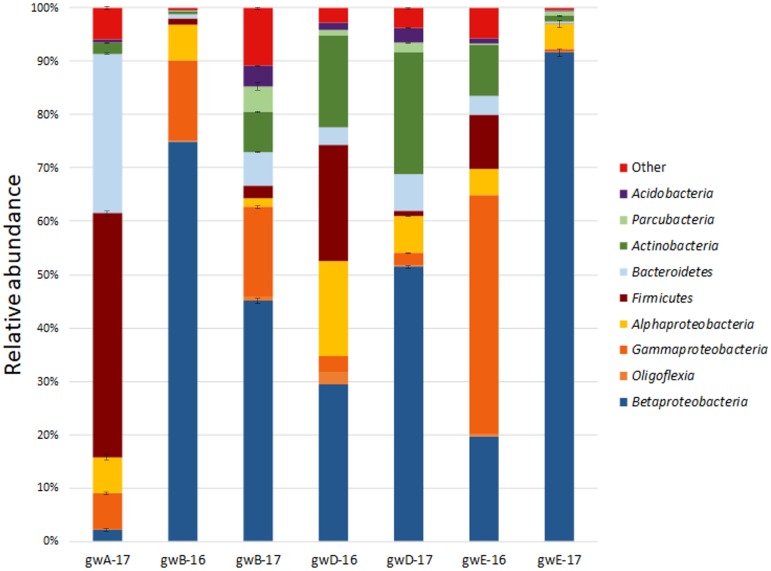
Taxonomic classification of bacterial 16S rRNA gene fragments in the libraries from groundwater samples at the phylum level (at the class level for *Proteobacteria*) using the RDP classifier.

**FIGURE 3:**
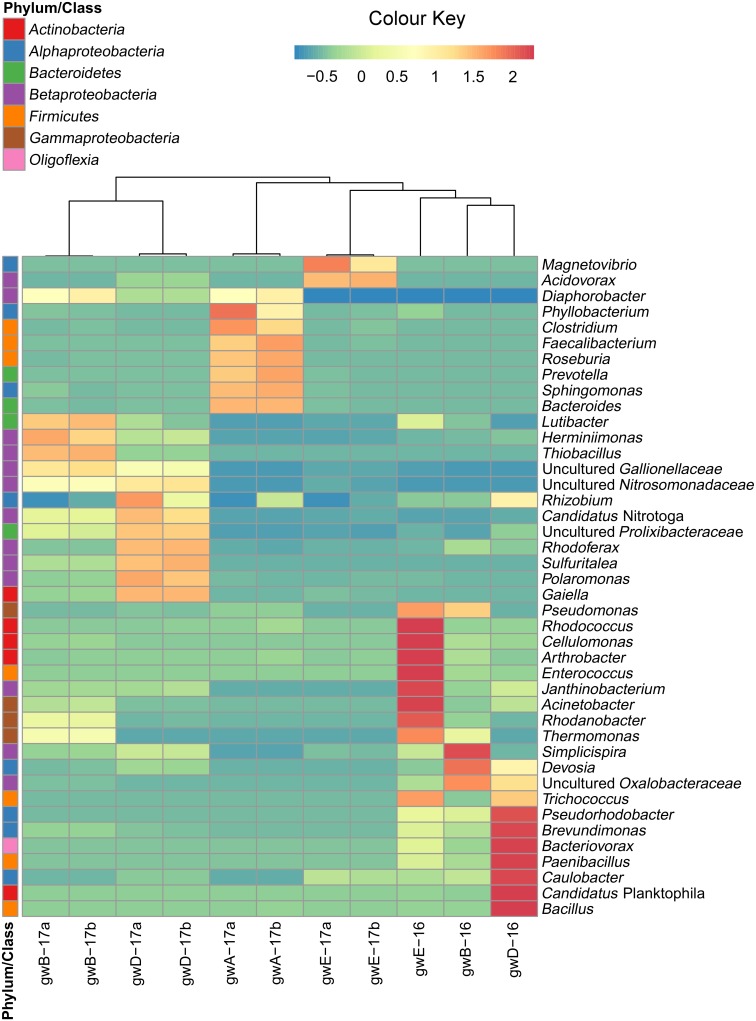
Heatmap analysis of the dominant genera distribution in seven groundwater samples. The double hierarchical dendrogram shows microbial distribution in these samples. The relative values for microbial genera are marked by colors from green to red designating the least abundant to most abundant. Abundance is expressed as the number of targeted sequences to the total number of sequences from each groundwater sample.

Taxonomic distribution of the bacterial 16S rRNA gene fragments at the phylum level and for the *Proteobacteria* at the class level was summarized in **Figure 2**. All sequences were assigned to phyla as demonstrated in **Supplementary Figure S2** and **Supplementary Table S3**. In groundwater from well A located outside the contaminated zone, predominant taxa were *Firmicutes* (44–47.2%), *Bacteroidetes* (29–29.9%), and *Proteobacteria* (13.8–18.2%). In six contaminated groundwater samples, *Proteobacteria* predominated, accounting for 52.6 to 97% of total reads in the libraries. Among the *Proteobacteria* sequences, those of the *Beta-, Alpha-*, and *Gamma-proteobacteria* were the most numerous. The other phyla retrieved in the libraries from contaminated groundwater were *Actinobacteria* (0.7–22.9%), *Bacteroidetes* (0.3–6.9%), *Firmicutes* (0.1–21.8%), *Parcubacteria* (0.03–6.5%), *Acidobacteria* (0.05–4.1%), and *Oligoflexia* (0–2.3%). The most abundant genera represented by more than 1% reads in two libraries included *Acidovorax, Simplicispira, Thermomonas, Thiobacillus, Gaiella, Pseudomonas, Brevundimonas, Acinetobacter, Paenibacillus, Bacillus, Rhodanobacter*, Uncultured *Oxalobacteraceae*, and Uncultured *Gallionellaceae* (**Figure 3** and **Supplementary Figure S3** and **Supplementary Table S3**). Among minor component of the libraries were the sequences of *Chloroflexi, Cyanobacteria, Verrucomicrobia, Gemmatimonadetes, Saccharibacteria, Planctomycetes*, and *Ignavibacteriae* (NCBI SRA No. SRP119496).

### Biodiversity of Enrichment Cultures of Denitrifying Bacteria

Enrichment cultures of denitrifying bacteria obtained by inoculation of the media containing milk whey and nitrate with groundwater collected in 2016 from wells B, D, and E (enrichments enrB, enrD, and enrE, respectively), were characterized by considerably lower diversity compared to groundwater samples from the relevant wells. In the libraries enrB, enrD, and enrE, *Proteobacteria* sequences constituted almost 100% (**Supplementary Table S3**). Selective conditions promoted increased shares of denitrifying *Rhizobium* spp. (*Alphaproteobacteria*) in enrichment cultures (32.7, 86.7, and 2.5% of the libraries enrE, enrD, and enrB, respectively). In the enrB enrichment, *Alphaproteobacteria* of the genus *Devosia* were predominant (77.5%). While uncultured *Betaproteobacteria* of the family *Oxalobacteraceae* were predominant in groundwater samples gwB-16 and gwD-16, they were not revealed in the relevant enrichments. *Gammaproteobacteria* of the genera *Thermomonas* and *Pseudomonas* were revealed both in enrichments enrB and enrE and in the corresponding groundwater samples. The minor components (below 0.3% of the sequences) were *Actinobacteria* in enrB, and enrE.

### Isolation and Identification of Pure Cultures of Aerobic Organotrophic and Denitrifying Bacteria From Groundwater and Detection of the *nirS* and *nirK* Genes

**FIGURE 4:**
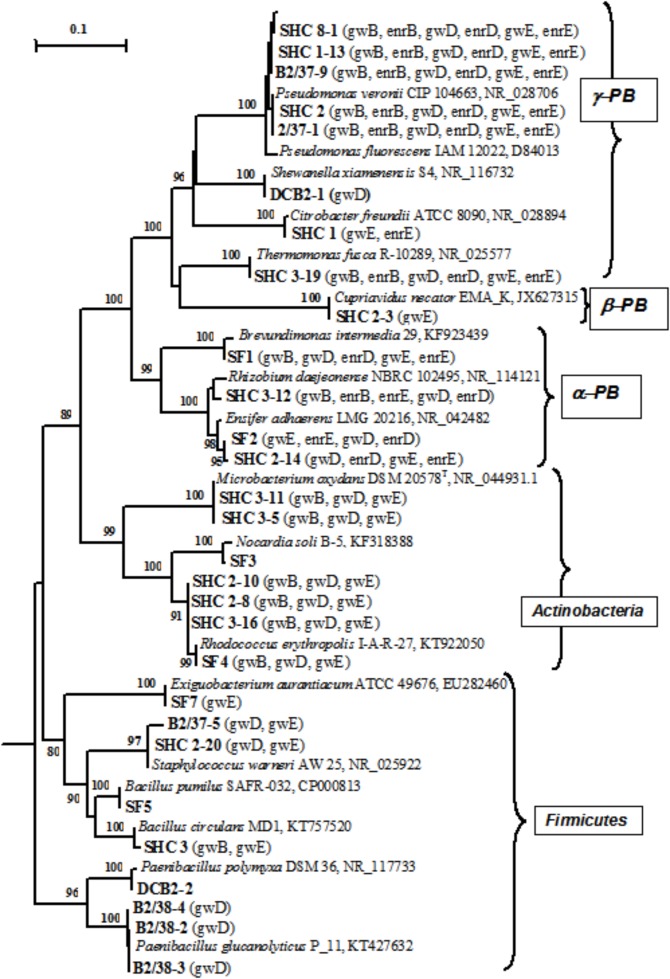
Phylogenetic tree of the 16S rRNA genes of pure cultures isolated from groundwater (shown in boldface). The libraries containing the OTUs (phylotypes) closely related to the 16S rRNA genes of the isolates (over 98% similarity) are shown in parentheses. The scale shows the evolutionary distance corresponding to five nucleotide replacements per 100 nucleotides. The numerals indicate the branching order determined by bootstrap analysis of 100 alternative trees (the values above 80 were accepted as significant). Accession numbers of 16S rRNA genes of isolated strains are given in **Supplementary Table S3**. *α-PB, Alphaproteobacteria; β-PB, Betaproteobacteria*, and *γ-PB, Gammaproteobacteria.*

Thirty-three pure cultures of aerobic organotrophic bacteria were isolated from groundwater samples. Since the 16S rRNA genes of the isolates exhibited 98–100% similarity to GenBank sequences, species identification of members of the following genera was possible: *Brevundimonas, Rhizobium, Ensifer* (*Alphaproteobacteria*); *Cupriavidus* (*Betaproteobacteria*); *Pseudomonas, Shewanella, Citrobacter, Thermomonas* (*Gammaproteobacteria*); *Microbacterium, Nocardia, Rhodococcus* (*Actinobacteria*); *Bacillus, Paenibacillus, Staphylococcus*, and *Exiguobacterium* (*Firmicutes*) (**Figure 4** and **Supplementary Table S4**). Among the 33 isolated strains, related phylotypes (at least 98% similarity) of 27 strains were revealed in the 16S rRNA gene libraries of groundwater microorganisms or in denitrifying enrichments.

Nitrite reduction to nitric oxide, which is catalyzed by NirS (cytochrome cd1) encoded by the *nirS* gene, or by NirK (copper-containing nitrite reductase) encoded by the *nirK* gene (dissimilatory nitrite reductase) is known to be the key stage of denitrification. Nitrite reductase genes are often used as molecular markers of denitrification in microbial communities (Hallin and Lindgren, 1999; Green et al., 2012). Using DNA from pure cultures and a number of primers (**Supplementary Table S1**) resulted in detection of *nirS* and/or *nirK* in 17 strains (**Supplementary Table S4**). The results of dinitrogen detection in the gas phase and detection of *nirS* and *nirK* with different primers did not always correlate. The absence of N_2_ release by the cultures for which nitrite reductase genes were detected may be due to incomplete denitrification with production of gaseous products other than dinitrogen (nitric oxide or nitrous oxide). Nine isolates reduced nitrate to nitrite, and 13 strains carried out complete denitrification with production of molecular nitrogen (**Supplementary Table S4**).

Growth of *Pseudomonas veronii* SHC 8-1, *Ensifer adhaerens* SHC 2-14, and *Thermomonas fusca* SHC 3-19 in the medium with acetate and nitrate resulted in nitrate ion concentration decrease from 1000 to 80–220 mg l^-1^ and Eh decrease from +140…+110 to +40…+50 mV (**Supplementary Figure S4**). Members of the genera *Thermomonas* and *Pseudomonas*, which predominated in groundwater from wells B and E, were studied in more detail. Strain *P. veronii* SHC 8-1 grew within a broad temperature range, from 5 to 35°C, with the optimum at 28–33°C, at 0 to 5% NaCl (optimum at 0% NaCl), and pH from 6.0 to 8.7 (pH optimum 7.7). Strain SHC 8-1 carried out denitrification with dinitrogen production using a broad range of substrates (acetate, methanol, ethanol, sucrose, fructose, glucose, milk whey, and yeast hydrolysate). Strain *Thermomonas fusca* SHC 3-19 grew at temperatures from 15 to 33°C (optimum at 28–33°C), at 0 to 3% NaCl in the medium (optimum at 0–0.5% NaCl), and at pH from 6 to 8.9 (pH optimum 7.2–8.0).

### *In situ* Bioremediation of Groundwater From Nitrate

**FIGURE 5:**
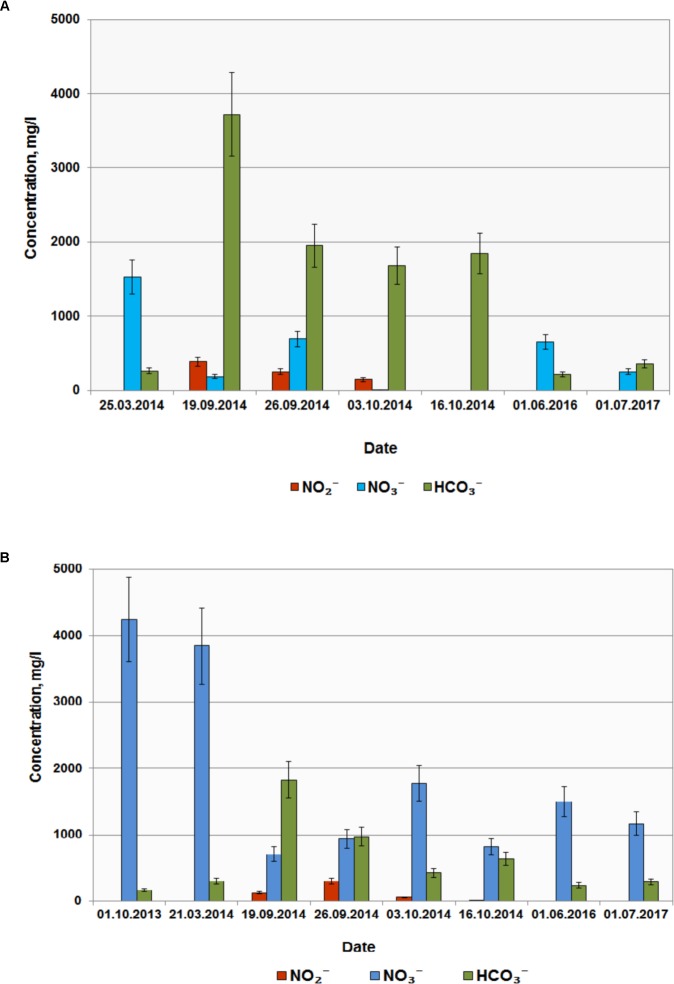
Concentrations of ${\mathrm{HCO}}_{3}^{-}$, ${\mathrm{NO}}_{3}^{-}$, and ${\mathrm{NO}}_{2}^{-}$ in groundwater from wells D **(A)** and B **(B)** before and after injection of organic substrates into the subsurface horizon.

Based on the results of laboratory studies, *in situ* trial of the biotechnological process for nitrate removal from groundwater was carried out at wells B and D with initial concentrations of 3840 and 1530 mg ${\mathrm{NO}}_{3}^{-}$ per 1 L groundwater, respectively. Groundwater samples (2–3 m^3^) were collected in metal cisterns equipped with stirrers. Water from well B was supplemented with milk whey and sodium acetate (31.0 L and 2.1 kg per 1 m^3^, respectively); water from well D was supplemented with 18 L/m^3^ and 7.0 kg/m^3^, respectively. These solutions were then injected back into the wells (single-well push-pull test). Aliquots (1 m^3^) were collected at regular intervals during 60 days and pumped back into the well after chemical analysis. After 10 days nitrate concentrations in wells B and D decreased below MPC (45 mg l^-1^); nitrite concentration increased up to 500 mg l^-1^, and complete nitrite removal from groundwater was observed after 20 and 30 days, respectively (**Figures 5A,B**). In the course of the experiment groundwater Eh decreased from 100…120 to -40 mV, which may promote reduction of redox-sensitive metals, formation of low-mobility compounds with poor water solubility, and prevent their propagation in groundwater. When injection of organic substrates was finished, nitrate- and radionuclide-contaminated groundwater returned to the zone of experimental wells (**Figure 5** and **Table 1**).

### Bacterial Community Composition and Geochemical Parameters of Groundwater

**FIGURE 6:**
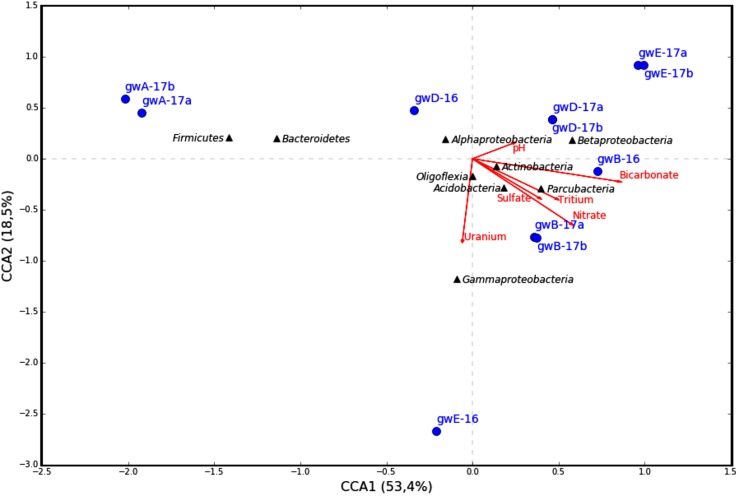
Canonical correspondence analysis (CCA) showing the correlation between bacterial diversity at phylum or class level in the 16S rRNA gene libraries of the microorganisms from groundwater and geochemical parameters of the groundwater samples.

**FIGURE 7:**
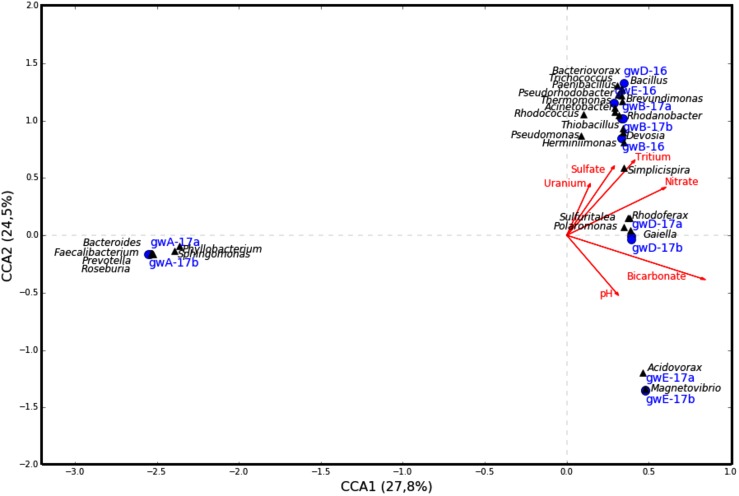
Canonical correspondence analysis showing the correlation between bacterial diversity at the genus level and geochemical parameters of the groundwater samples.

The similarity between microbial communities in seven studied groundwater samples was determined. The results of principal component analysis based on the 16S rRNA gene sequences from groundwater samples are shown on the plot matrix (**Supplementary Figure S5**). This figure demonstrates that microbial community composition in groundwater from well A located outside the zone of waste dispersion was essentially different from microbial composition in all studied contaminated groundwater samples. The six geochemical parameters of groundwater (nitrate, uranium, tritium, pH, sulfate, and bicarbonate) and abundance of the 16S rRNA gene sequences at the phylum or class level (**Figure 6**) and at the genus level (**Figure 7**) from groundwater libraries were used for CCA. CCA supported the conclusion of principal component analysis and additionally showed the difference between microbial communities of uncontaminated and contaminated groundwater. The studied geochemical parameters had probably no effect on the composition of the microbial community from well A (gwA-17a and gwA-17b libraries). The presence of members of the phylum *Firmicutes*, as well as of *Bacteroides* and the fecal bacteria of the genus *Faecalibacterium*, as well as elevated level of TOC (9.93 mg l^-1^, **Table 1**), indicated mixing of groundwater and surface water in the area of well A. Nitrate and concomitant contaminants significantly linked to the overall microbial communities of groundwater from B, D, and E wells and determined the presence of members of the genera *Acidivorax, Simplicispira, Pseudomonas, Brevundimonas, Thermomonas, Acinetobacter*, capable of denitrification with dinitrogen release or of nitrate reduction to nitrite (**Figure 7**) (Elomari et al., 1996; Mergaert et al., 2003; Grabovich et al., 2006; Choi et al., 2010).

These results indicate that a unique microbial community formed in groundwater at the area of a suspended surface repository for liquid RAW, which differed from the community of natural groundwater.

Cultured aerobic organotrophic and nitrifying bacteria, as well as anaerobic fermenting, iron-reducing, and denitrifying bacteria were revealed in groundwater samples (**Supplementary Figure S1**). High redox potential (Eh ∼150 mV) of groundwater probably prevented development of anaerobic methanogenic and sulfate-reducing prokaryotes. Culture-based techniques revealed them only in few samples in numbers at the sensitivity limit of the method (≤10 cells/mL).

These data are in agreement with the results of high-throughput sequencing of the V3–V4/V4 regions of the 16S rRNA genes of the microbial community, which revealed low presentation of the 16S rRNA genes of methanogenic archaea and sulfate-reducing bacteria in the libraries based on the DNA of groundwater microorganisms. Out of 12 genera with the 16S rRNA genes predominant in the libraries from contaminated groundwater (>5% sequences in the library), 11 genera included bacteria capable of complete denitrification with formation of gaseous products (NO, N_2_O, and/or N_2_) or of nitrate reduction to nitrite (**Supplementary Table S4**).

While the sequences of uncultured members of the family *Oxalobacteraceae*, and bacteria of the genera *Acidovorax, Simplicispira* (*Betaproteobacteria*), *Brevundimonas* (*Alphaproteobacteria*), *Pseudomonas, Thermomonas*, and *Acinetobacter* (*Gammaproteobacteria*) were found in all seven libraries from groundwater, their share was especially high in the libraries from contaminated groundwater (**Figure 3** and **Supplementary Table S3**).

For example, in the libraries from the most contaminated groundwater (well B), the sequences of the phylum *Proteobacteria* (97, 66.3, and 63.5% in gwB-16, gwB-17a and gwB-17b, respectively) belonged to the *Betaproteobacteria* (75, 45.7, and 44%), including uncultured *Oxalobacteraceae, Simplicispira*, and *Thiobacillus*, which has denitrifying members of the genus, and aerobic bacteria *Herminiimonas*, which occur in metal-contaminated habitats and in fresh subterranean water (Grabovich et al., 2006; Muller et al., 2006). The sequences of *Gammaproteobacteria* (15.1, 17.4, and 16.3%) belonged to members of the genera *Thermomonas* and *Pseudomonas* (**Figure 3** and **Supplementary Table S3**). The sequences of *Alphaproteobacteria* in the gwB-16 library belonged to aerobic bacteria of the genus *Devosia* (4.3%) which are widespread in soils and aquatic ecosystems (Zhang et al., 2012). Thus, long-term contamination with nitrate ions resulted in enrichment of the bacterial community with denitrifying bacteria.

In the libraries from the well D groundwater, together with the sequences of the *Simplicispira*, uncultured *Oxalobacteraceae, Acidovorax*, and *Brevundimonas* numerous *Firmicutes* sequences were present (21.8% in gwD-16), including those of the genera *Paenibacillus* (9.5%) and *Bacillus* (7.0%); and *Actinobacteria* (17.4%) sequences belonging to *Candidatus* Planktophila, which is widespread in freshwater habitats (Jezbera et al., 2009), and sequences of novel and rare bacterial genus *Gaiella. Gaiellaceae*, a recently described family, was originally found in a deep water horizon, and sequences from this family have been found in soil, volcanic soil, thermal springs, and marine ascidians (Albuquerque et al., 2011).

The composition of microbial communities from the well E groundwater, sampled in 2016 and 2017 exhibited most pronounced differences. In gwE-17a and gwE-17b libraries, 86.8–89% sequences belonged to *Acidovorax* (**Figures 2, 3, 7**, **Table 2** and **Supplementary Figure S5** and **Supplementary Table S3**). This implies the necessity for analysis of dynamics of the groundwater communities in order to reveal the major functional groups.

Our results are similar to those obtained earlier in analysis of clone libraries of the microorganisms from nitrate-and uranium-contaminated subsurface sediments at the Oak Ridge Field Research Center (Akob et al., 2007). The sequences related to nitrate-reducing bacteria, such as members of the *Proteobacteria* (including the genera *Sphingomonas, Acidovorax, Acinetobacter, Alcaligenes*, and *Ralstonia*), showed a high relative abundance in the total (28%) and metabolically active (43%) fractions of the microbial community. Investigation of microcosms from nitrate- and radionuclide-contaminated groundwater at OR-FRC revealed that the diversity and activity of nitrate- and Fe(3+)-reducing microbial populations catalyzing U(VI) reduction and immobilization in the sediments depended directly on the introduced electron donors (Akob et al., 2008).

Phylogenetic diversity of enrichment cultures of denitrifying bacteria obtained in the medium with milk whey and nitrate was considerably lower than the diversity of groundwater microorganisms. Selective conditions resulted in the loss of uncultured *Oxalobacteraceae* and *Candidatus* Planktophila, which were revealed in groundwater by molecular techniques (**Supplementary Table S3**).

Denitrifying microcosms from contaminated groundwater were adapted to the temperature, pH, and nitrate concentration of this environment, exhibited high catabolic potential, and reduced nitrate to dinitrogen when grown on a broad range of organic substrates. Preferable substrates were milk whey, sucrose, and acetate (**Supplementary Table S2**). Pumping of milk whey and acetate into the subterranean horizon stimulated denitrification and local removal of nitrate ions from groundwater. Groundwater analysis 1.5 years after the biotechnological treatment showed that contaminated water returned to the zone of the wells B, E, and D (**Figure 5** and **Table 1**).

Pure cultures of aerobic organotrophs, iron-reducing, and denitrifying bacteria isolated from groundwater belonged to 15 genera and 22 species. The 16S rRNA gene sequences similar to those of the isolated strains were also revealed by high-throughput sequencing in groundwater samples and denitrifying enrichment cultures. Nitrite reductase genes *nirS* and *nirK* were identified in denitrifying bacteria of the genera *Pseudomonas, Thermomonas*, and *Ensifer*, as well as in other strains with the help of specific primers (**Supplementary Table S4**). The products obtained by PCR amplification of the *nirS* and *nirK* genes were heterogeneous, which prevented direct determination of their sequences. We found that pure cultures of bacteria isolated from groundwater caused Eh of the medium to decrease by 60–85 mV in the course of denitrification, which may promote development of anoxic conditions favorable for uranium reduction and its decreased migration (**Supplementary Figure S4**). Since uranium concentration in the studied groundwater was low, investigation of microbial action on radionuclides was beyond the scope of the present work.

Denitrifying bacteria isolated from nitrate- and radionuclide-contaminated groundwater and sediments at Oak Ridge included members of the genera *Afipia, Hyphomicrobium, Rhodanobacter, Intrasporangium*, and *Bacillus* (Green et al., 2010, 2012).

In the studied subterranean horizon, members of the genera *Simplicispira, Rhizobium, Pseudomonas, Brevundimonas, Ensifer, Thermomonas, Acidovorax*, and *Rhodanobacter* are probably responsible for nitrate reduction. Using for phylogenetic analysis the RNA of groundwater microorganisms, rather than DNA, will provide for determination of the metabolically active bacteria involved in nitrate removal from groundwater *in situ*.

## Conclusion

The results of chemical and radiochemical analysis of groundwater samples in the area of a suspended surface repository for liquid RAW indicated technogenic contamination with nitrate, sulfate, and bicarbonate ions, as well as with tritium and strontium. Abundance of nitrate in the studied groundwater provided selective pressure in favor of denitrifying bacteria adapted to this environment. When supplied with carbon sources and electron donors (milk whey or acetate), denitrifying populations were able to carry out *in situ* removal of nitrate ions. Denitrifying populations included members of bacterial genera *Simplicispira, Rhizobium, Pseudomonas, Brevundimonas, Ensifer, Thermomonas, Acidovorax*, and *Rhodanobacter*, which were revealed by molecular and/or culture-based techniques. The results obtained provide information on the composition of the subterranean microbial community in the zone of nitrate and radionuclide contamination and indicate the possibility of successful groundwater remediation by nitrate removal.

## Author Contributions

AN, EZ, and AS contributed to field work and performed radiochemical analysis. TB and DS estimated numbers of microorganisms and obtained enrichment and pure cultures. DS, AP, and AM obtained DNA, performed molecular studies and high-throughput sequencing. TT and DG performed phylogenetic and statistical analyses. TN, AS, and AN designed the work. TN, AS, TB, DS, DG, TT, AP, EZ, AM, and AN analyzed the data, wrote and approved the manuscript.

## Conflict of Interest Statement

The authors declare that the research was conducted in the absence of any commercial or financial relationships that could be construed as a potential conflict of interest.

## Footnotes

1 http://www.esd.ornl.gov/orifrc/

2 http://rdp.cme.msu.edu

3 http://jwbrown.mbio.ncsu.edu/BioEdit/bioedit.html

4 https://github.com/jstjohn/SeqPrep

5 http://www.ncbi.nlm.nih.gov/blast
