# Supplementary material for: Microbial Community and in situ Bioremediation of Groundwater by Nitrate Removal in the Zone of a Radioactive Waste Surface Repository
Source: Front Microbiol. 2018 Aug 23;9:1985. doi: 10.3389/fmicb.2018.01985 (PMC6115527; doi:10.3389/fmicb.2018.01985)
Supplement: Supplementary file 1 [file Data_Sheet_1.pdf]

# *Supplementary Material*

## Microbial Community and *in situ* Bioremediation of Groundwater by Nitrate Removal in the Zone of a Radioactive Waste Surface Repository

**Alexey V. Safonov[1, 5], Tamara L. Babich[2], Diyana S. Sokolova[2], Denis S. Grouzdev[3], Tatiyana P. Tourova[2], Andrey B. Poltaraus[4], Elena V. Zakharova[1, 5], Alexander Y. Merkel[2], Alexander P. Novikov[5], Tamara N. Nazina[5, 2, *]**

*[1]Frumkin Institute of Physical Chemistry and Electrochemistry, Russian Academy of Sciences, Moscow, Russian Federation, [2]Winogradsky Institute of Microbiology, Research Center of Biotechnology, Russian Academy of Sciences, Moscow, Russian Federation, [3]Institute of Bioengineering, Research Center of Biotechnology of the Russian Academy of Sciences, Moscow, Russian Federation, [4]Engelhardt Institute of Molecular Biology, Russian Academy of Sciences, Moscow, Russian Federation, [5]V.I. Vernadsky Institute of Geochemistry and Analytical Chemistry of Russian Academy of Sciences, Moscow, Russian Federation*

* Correspondence:
**Dr. Tamara N. Nazina**
e-mail: nazina@inmi.ru

## Supplementary Tables

**Supplementary Table S1 |** Primer sequences and positions used to amplify fragments from *nirK* and *nirS* nitrite reductase genes

| Primer | Primer sequence (5′-3′) | Primer position | Reference |
|---|---|---|---|
| nirK1F | GGM ATG GTK CCS TGG CA | 526–542 | Braker et al., 1998 |
| nirK5R | GCC TCG ATC AGR TTR TGG | 1040–1023 | |
| nirS1F | CCT AYT GGC CGC CRC ART | 763–780 | |
| nirS6R | CGT TGA ACT TRC CGG T | 1653–1638 | |
| nirK517F | TTYGTSTAYCACTGCGCVCC | 517 | Chen et al., 2010 |
| nirK1055R | GCYTCGATCAGRTTRTGGTT | 1055 | Michotey et al., 2000 |
| nirS(cd3aF) | GT(C/G) AAC GT(C/G) AAG GA(A/G) AC(C/G) GG | 916–935 | |
| nirS(R3cd) | GA(C/G) TTC GG(A/G) TG(C/G) GTC TTG A | 1322–1341 | Throbäck et al., 2004 |

**Supplementary Table S2 |** Nitrate removal and nitrite accumulation by the microcosms of groundwater samples from wells A, B, and C, supplemented with various organic substrates (15 days of incubation at 22°C)

| Substrate | Microcosm A | | Microcosm B | | Microcosm C | |
|---|---|---|---|---|---|---|
| | $NO_3^-$ | $NO_2^-$ | $NO_3^-$ | $NO_2^-$ | $NO_3^-$ | $NO_2^-$ |
| | Initial concentration, $mg \cdot l^{-1}$ | | | | | |
| | 1000 | 0 | 4239 | 0 | 3280 | 0 |
| | Residual concentration, $mg \cdot l^{-1}$ | | | | | |
| Methanol | 889.8 | 20 | 2980.0 | 40 | 1793.7 | 70 |
| Ethanol | 911.6 | 20 | 1628.4 | 70 | 1928.6 | 40 |
| Oxalate | 590.0 | 50 | 1793.7 | 0 | 1133.1 | 50 |
| Acetate | 474.6 | 100 | 1793.7 | 20 | 1374.9 | 60 |
| Lactate | 229.8 | 70 | 2839.4 | 50 | 1278.7 | 60 |
| Glucose | 788.5 | 30 | 2908.9 | 70 | 1928.6 | 30 |
| Sucrose | 788.5 | 30 | 2771.7 | 50 | 1668.2 | 70 |
| Milk whey | 17.7 | 10 | 94.0 | 60 | 21.5 | 70 |
| Kefir whey | 50.1 | 70 | 2176.4 | 100 | 1079.7 | 100 |
| Yeast extract | 292.7 | 10 | 67.0 | 60 | 30.2 | 10 |

**Supplementary Table S3.** Diversity of the dominant phylotypes of the *Bacteria* domain in 16S rRNA gene libraries of the microorganisms from groundwater and denitrifying enrichments

| Taxonomic group | Share of the number of sequences in the library* from well, % | | | | | | | | | | | | | |
|---|---|---|---|---|---|---|---|---|---|---|---|---|---|---|
| | A | | B | | | | D | | | | E | | | |
| | gwA-17a | gwA-17b | gwB-16 | gwB-17a | gwB-17b | enrB | gwD-16 | gwD-17a | gwD-17b | enrD | gwE-16 | gwE-17a | gwE-17b | enrE |
| ***Proteobacteria*** | 18.20 | 13.76 | 96.97 | 66.32 | 63.51 | 99.64 | 52.64 | 62.00 | 60.88 | 99.91 | 71.20 | 96.79 | 96.93 | 99.90 |
| ***Alphaproteobacteria*** | 7.38 | 5.49 | 6.76 | 1.51 | 1.54 | 80.10 | 17.80 | 7.09 | 6.73 | 89.60 | 4.84 | 5.82 | 3.67 | 36.50 |
| *Brevundimonas* | 0.00 | 0.00 | 0.96 | 0.42 | 0.45 | 0.00 | 7.00 | 0.23 | 0.13 | 0.53 | 1.66 | 0.04 | 0.05 | 0.00 |
| *Caulobacter* | 0.00 | 0.00 | 0.11 | 0.01 | 0.01 | 0.00 | 0.57 | 0.04 | 0.04 | 0.64 | 0.08 | 0.10 | 0.08 | 2.98 |
| *Devosia* | 0.00 | 0.00 | 4.28 | 0.12 | 0.14 | 77.54 | 2.47 | 0.49 | 0.45 | 1.32 | 0.30 | 0.02 | 0.02 | 0.44 |
| *Magnetovibrio* | 0.00 | 0.00 | 0.00 | 0.00 | 0.00 | 0.00 | 0.00 | 0.01 | 0.03 | 0.00 | 0.00 | 2.92 | 1.96 | 0.00 |
| *Phyllobacterium* | 3.31 | 1.91 | 0.01 | 0.07 | 0.04 | 0.00 | 0.00 | 0.01 | 0.01 | 0.00 | 0.20 | 0.00 | 0.02 | 0.00 |
| *Pseudorhodobacter* | 0.00 | 0.00 | 0.61 | 0.00 | 0.00 | 0.00 | 2.65 | 0.00 | 0.00 | 0.00 | 0.75 | 0.00 | 0.00 | 0.00 |
| *Rhizobium* | 0.00 | 0.04 | 0.02 | 0.00 | 0.01 | 2.47 | 0.09 | 0.13 | 0.06 | 86.75 | 0.02 | 0.00 | 0.01 | 32.70 |
| *Sphingomonas* | 1.30 | 1.37 | 0.00 | 0.07 | 0.01 | 0.00 | 0.00 | 0.02 | 0.02 | 0.00 | 0.00 | 0.03 | 0.00 | 0.00 |
| ***Betaproteobacteria*** | 2.61 | 1.89 | 75.00 | 45.70 | 44.00 | 12.90 | 29.50 | 51.80 | 51.10 | 10.10 | 19.60 | 90.30 | 92.70 | 26.70 |
| *Acidovorax* | 0.14 | 0.18 | 0.02 | 0.21 | 0.20 | 0.35 | 0.50 | 9.65 | 10.17 | 1.22 | 0.23 | 86.81 | 89.05 | 0.44 |
| *Candidatus* Nitrotoga | 0.00 | 0.00 | 0.00 | 0.50 | 0.48 | 0.00 | 0.03 | 1.20 | 1.07 | 0.00 | 0.00 | 0.01 | 0.04 | 0.00 |
| *Diaphorobacter* | 0.07 | 0.08 | 0.00 | 0.07 | 0.08 | 8.11 | 0.00 | 0.03 | 0.03 | 0.00 | 0.00 | 0.00 | 0.00 | 0.02 |
| *Herminiimonas* | 0.00 | 0.00 | 0.09 | 1.10 | 0.96 | 0.00 | 0.12 | 0.27 | 0.31 | 0.00 | 0.07 | 0.00 | 0.02 | 0.00 |
| *Janthinobacterium* | 0.00 | 0.00 | 0.08 | 0.11 | 0.12 | 0.00 | 0.22 | 0.12 | 0.15 | 0.00 | 0.99 | 0.00 | 0.00 | 0.00 |
| *Polaromonas* | 0.11 | 0.02 | 0.02 | 0.36 | 0.41 | 0.00 | 0.07 | 3.96 | 3.62 | 0.00 | 0.09 | 0.05 | 0.10 | 0.02 |
| *Rhodoferax* | 0.11 | 0.00 | 1.79 | 0.77 | 0.87 | 0.00 | 0.91 | 9.46 | 9.57 | 0.00 | 0.40 | 0.30 | 0.30 | 0.00 |
| *Simplicispira* | 0.10 | 0.03 | 39.41 | 4.52 | 4.65 | 1.93 | 1.69 | 9.02 | 8.78 | 0.00 | 8.73 | 2.48 | 2.44 | 0.07 |
| *Sulfuritalea* | 0.00 | 0.00 | 0.00 | 0.29 | 0.29 | 0.00 | 0.01 | 1.45 | 1.52 | 0.00 | 0.01 | 0.00 | 0.00 | 0.00 |
| *Thiobacillus* | 0.01 | 0.01 | 0.00 | 21.54 | 22.23 | 0.00 | 0.00 | 1.96 | 1.81 | 0.00 | 0.00 | 0.02 | 0.04 | 0.00 |
| Uncult.*** *Nitrosomonadaceae* | 0.00 | 0.00 | 0.00 | 1.02 | 1.02 | 0.00 | 0.00 | 1.34 | 1.39 | 0.00 | 0.00 | 0.10 | 0.05 | 0.00 |

| | | | | | | | | | | | | | | |
|---|---|---|---|---|---|---|---|---|---|---|---|---|---|---|
| Uncult. *Gallionellaceae* | 0.01 | 0.00 | 0.00 | 7.48 | 7.95 | 0.00 | 0.01 | 5.25 | 5.03 | 0.00 | 0.03 | 0.28 | 0.40 | 0.00 |
| Uncult. *Oxalobacteraceae* | 0.02 | 0.00 | 28.31 | 0.96 | 1.02 | 0.00 | 21.62 | 0.30 | 0.23 | 0.00 | 4.54 | 0.02 | 0.03 | 0.00 |
| ***Gammaproteobacteria*** | 7.71 | 5.94 | 15.10 | 17.40 | 16.30 | 6.64 | 2.87 | 2.26 | 2.11 | 0.21 | 44.90 | 0.66 | 0.55 | 36.70 |
| *Acinetobacter* | 0.02 | 0.05 | 0.40 | 1.30 | 1.62 | 0.00 | 1.52 | 0.15 | 0.16 | 0.00 | 10.30 | 0.00 | 0.00 | 0.00 |
| *Pseudomonas* | 0.53 | 0.55 | 5.26 | 0.16 | 0.15 | 2.99 | 0.03 | 0.34 | 0.30 | 0.05 | 6.17 | 0.00 | 0.01 | 31.81 |
| *Rhodanobacter* | 0.02 | 0.02 | 0.26 | 1.22 | 1.17 | 0.23 | 0.00 | 0.02 | 0.03 | 0.00 | 3.78 | 0.00 | 0.00 | 0.00 |
| *Thermomonas* | 0.00 | 0.00 | 8.90 | 10.99 | 11.24 | 3.39 | 0.23 | 0.00 | 0.00 | 0.11 | 23.03 | 0.01 | 0.02 | 4.49 |
| ***Oligoflexia*** | 0.00 | 0.00 | 0.09 | 0.67 | 0.71 | 0.00 | 2.35 | 0.30 | 0.36 | 0.00 | 0.42 | 0.00 | 0.00 | 0.00 |
| *Bacteriovorax* | 0.00 | 0.00 | 0.08 | 0.00 | 0.00 | 0.00 | 1.71 | 0.00 | 0.00 | 0.00 | 0.38 | 0.00 | 0.00 | 0.00 |
| ***Actinobacteria*** | 1.98 | 2.35 | 0.73 | 7.97 | 7.22 | 0.32 | 17.40 | 22.90 | 22.90 | 0.00 | 9.48 | 1.43 | 0.95 | 0.02 |
| *Arthrobacter* | 0.01 | 0.04 | 0.11 | 0.00 | 0.01 | 0.00 | 0.00 | 0.02 | 0.01 | 0.00 | 1.24 | 0.00 | 0.01 | 0.00 |
| *Candidatus* Planktophila | 0.00 | 0.00 | 0.05 | 0.01 | 0.01 | 0.00 | 14.03 | 0.02 | 0.01 | 0.00 | 0.23 | 0.00 | 0.00 | 0.00 |
| *Cellulomonas* | 0.00 | 0.00 | 0.13 | 0.04 | 0.06 | 0.26 | 0.05 | 0.00 | 0.01 | 0.00 | 1.43 | 0.00 | 0.00 | 0.00 |
| *Gaiella* | 0.01 | 0.00 | 0.02 | 1.60 | 1.85 | 0.00 | 0.11 | 16.88 | 16.75 | 0.00 | 0.13 | 0.67 | 0.41 | 0.00 |
| *Rhodococcus* | 0.06 | 0.22 | 0.09 | 0.01 | 0.01 | 0.00 | 0.10 | 0.02 | 0.03 | 0.00 | 3.71 | 0.00 | 0.00 | 0.00 |
| ***Firmicutes*** | 44.00 | 47.20 | 1.17 | 2.35 | 2.35 | 0.00 | 21.80 | 0.88 | 0.97 | 0.00 | 10.10 | 0.09 | 0.08 | 0.00 |
| *Bacillus* | 0.02 | 0.07 | 0.05 | 0.00 | 0.00 | 0.00 | 7.07 | 0.00 | 0.00 | 0.00 | 0.05 | 0.00 | 0.00 | 0.00 |
| *Clostridium* | 1.13 | 0.90 | 0.00 | 0.00 | 0.01 | 0.00 | 0.00 | 0.00 | 0.00 | 0.00 | 0.00 | 0.00 | 0.02 | 0.00 |
| *Enterococcus* | 0.02 | 0.00 | 0.17 | 0.00 | 0.00 | 0.00 | 0.06 | 0.00 | 0.00 | 0.00 | 3.35 | 0.00 | 0.00 | 0.00 |
| *Faecalibacterium* | 12.10 | 14.04 | 0.00 | 0.04 | 0.04 | 0.00 | 0.00 | 0.04 | 0.04 | 0.00 | 0.00 | 0.01 | 0.02 | 0.00 |
| *Paenibacillus* | 0.00 | 0.00 | 0.72 | 0.00 | 0.00 | 0.00 | 9.51 | 0.00 | 0.00 | 0.00 | 1.90 | 0.00 | 0.00 | 0.00 |
| *Roseburia* | 3.03 | 3.31 | 0.00 | 0.00 | 0.01 | 0.00 | 0.00 | 0.01 | 0.01 | 0.00 | 0.00 | 0.00 | 0.00 | 0.00 |
| *Trichococcus* | 0.00 | 0.00 | 0.08 | 0.00 | 0.00 | 0.00 | 1.81 | 0.00 | 0.00 | 0.00 | 2.09 | 0.00 | 0.00 | 0.00 |
| ***Bacteroidetes*** | 29.00 | 29.90 | 0.63 | 6.28 | 6.00 | 0.00 | 3.16 | 6.88 | 6.74 | 0.00 | 3.62 | 0.38 | 0.33 | 0.00 |
| *Bacteroides* | 12.03 | 12.04 | 0.00 | 0.05 | 0.02 | 0.00 | 0.00 | 0.03 | 0.04 | 0.00 | 0.00 | 0.04 | 0.02 | 0.00 |
| *Lutibacter* | 0.00 | 0.00 | 0.13 | 1.06 | 1.11 | 0.00 | 0.00 | 0.25 | 0.13 | 0.00 | 0.41 | 0.02 | 0.03 | 0.00 |
| *Prevotella* | 8.42 | 9.47 | 0.00 | 0.01 | 0.02 | 0.00 | 0.00 | 0.04 | 0.02 | 0.00 | 0.00 | 0.01 | 0.00 | 0.00 |
| Uncult. *Prolixibacteraceae* | 0.00 | 0.00 | 0.05 | 1.54 | 1.36 | 0.00 | 0.58 | 3.72 | 3.62 | 0.00 | 0.21 | 0.04 | 0.03 | 0.00 |

| | | | | | | | | | | | | | | |
|---|---|---|---|---|---|---|---|---|---|---|---|---|---|---|
| ***Acidobacteria*** | 0.34 | 0.71 | 0.05 | 3.59 | 4.12 | 0.00 | 1.35 | 2.41 | 3.00 | 0.00 | 0.94 | 0.17 | 0.27 | 0.14 |
| ***Parcubacteria*** | 0.00 | 0.00 | 0.03 | 2.99 | 6.55 | 0.00 | 0.90 | 2.05 | 1.78 | 0.00 | 0.19 | 0.57 | 0.83 | 0.00 |

*The taxonomic groups comprising >1% of the total number of *Bacteria* sequences are listed.

**0 corresponds <0.01.

***Uncult. designates Uncultured

**Supplementary Table S4 |** Taxonomic position of the strains isolated from groundwater of the radioactive waste repository area, their ability to carry out denitrification, and detection of the *nirS* and *nirK* genes

| Strain | Closest cultured strain, GenBank no. of the 16S rRNA sequence | Similarity of the 16S rRNA genes, % | Read length, bp | GenBank no. of the 16S rRNA sequence | $NO_3^-$ → $NO_2^-$ | $NO_3^-$ → $N_2$ | PCR detection of the *nirK* gene with primers: | | PCR detection of the *nirS* gene with primers: | |
|---|---|---|---|---|---|---|---|---|---|---|
| | | | | | | | nirK1F/ nirK5R | nirK517F/ nirK1055R | nirS1F/ nirS6R | nirS(cd3aF)/ nirS(R3cd) |
| | ***Alphaproteobacteria*** | | | | | | | | | |
| SF1 | *Brevundimonas intermedia* strain 29, KF923439 | 99 | 874 | MG051297 | + | - | - | - | - | - |
| SF14 | *Brevundimonas intermedia* strain 29, KF923439 | 99 | 1326 | | - | - | - | - | - | + |
| SHC 3-12 | *Rhizobium daejeonense* strain NBRC 102495, NR_114121 | 98 | 696 | MG051315 | + | + | + | - | - | + |
| SF2 | *Ensifer adhaerens* strain LMG 20216, NR_042482 | 99 | 1390 | MG051298 | - | + | + | - | - | - |
| SHC 2-14 | *Ensifer adhaerens* strain LMG 20216, NR_042482 | 100 | 642 | MG051317 | - | + | + | + | + | + |
| | ***Betaproteobacteria*** | | | | | | | | | |
| SHC 2-3 | *Cupriavidus necator* strain EMA_K, JX627315 | 99 | 638 | MG051310 | + | - | - | - | - | - |
| | ***Gammaproteobacteria*** | | | | | | | | | |
| SHC 5-3 | *Pseudomonas extremaustralis* strain 14-3, NR_114911 | 99 | 1034 | | + | + | + | - | - | + |
| SHC 1-17 | *Pseudomonas veronii* strain CIP 104663, NR_028706.1 | 100 | 635 | MG051319 | + | - | - | + | - | - |
| SHC 8-1 | *Pseudomonas veronii* strain CIP 104663, NR_028706.1 | 100 | 1395 | MG051309 | - | + | + | - | + | + |
| B2/37-9 | *Pseudomonas veronii* strain CIP 104663, NR_028706.1 | 99 | 439 | MG051305 | - | + | - | - | - | + |

| | | | | | | | | | | |
|---|---|---|---|---|---|---|---|---|---|---|
| SHC 2 | *Pseudomonas veronii* strain CIP 104663, NR_028706.1 | 99 | 619 | MG051323 | | | | | | |
| SHC 1-13 | *Pseudomonas mandelii* strain CIP 105273$^T$, NR_024902 | 99 | 645 | MG051316 | - | + | - | - | - | + |
| B2/37-1 | *Pseudomonas fluorescens* strain S15, KT223383 | 99 | 910 | MG051303 | - | + | - | - | - | - |
| DCB2-1 | *Shewanella xiamenensis* strain S4, NR_116732 | 100 | 558 | MG051295 | + | + | - | - | - | - |
| SHC 1 | *Citrobacter freundii* strain ATCC 8090, NR_028894 | 99 | 1160 | MG051322 | | | | | | |
| SHC 3-19 | *Thermomonas fusca* strain R-10289, NR_025577 | 99 | 601 | MG051320 | + | - | - | - | - | + |
| | ***Actinobacteria*** | | | | | | | | | |
| SHC 3-11 | *Microbacterium oxydans* strain DSM 20578$^T$, NR_044931.1 | 100 | 628 | MG051314 | - | - | - | - | - | - |
| SHC 3-5 | *Microbacterium oxydans* strain DSM 20578$^T$, NR_044931.1 | 100 | 739 | MG051311 | | | | | | |
| SF3 | *Nocardia soli* strain B-5, KF318388 | 99 | 1387 | MG051299 | + | - | - | - | - | - |
| SHC 2-10 | *Rhodococcus erythropolis* strain I-A-R-27, KT922050 | 100 | 1013 | MG051313 | - | + | - | - | - | + |
| SHC 2-8 | *Rhodococcus erythropolis* strain I-A-R-27, KT922050 | 99 | 713 | MG051312 | | | | | | |
| SHC 3-16 | *Rhodococcus erythropolis* strain I-A-R-27, KT922050 | 100 | 672 | MG051318 | - | + | - | + | - | + |
| SF4 | *Rhodococcus erythropolis* strain I-A-R-27, KT922050 | 100 | 1370 | MG051300 | - | + | - | - | - | - |
| SF13 | *Rhodococcus jialingiae* strain djl-6-2 | 99 | 1016 | | - | + | - | - | - | - |
| | ***Firmicutes*** | | | | | | | | | |
| SF7 | *Exiguobacterium aurantiacum*, strain ATCC 49676, EU282460 | 100 | 1404 | MG051302 | | | | | | |

| | | | | | | | | | | |
|---|---|---|---|---|---|---|---|---|---|---|
| B2/37-5 | *Staphylococcus pasteuri* strain ATCC 51129, NR_024669 | 99 | 478 | MG051304 | | | | | | |
| SHC 2-20 | *Staphylococcus warneri* strain AW 25, NR_025922 | 99 | 1035 | MG051321 | - | + | - | - | - | + |
| SF5 | *Bacillus pumilus* SAFR-032, CP000813 | 100 | 1490 | MG051301 | - | - | - | - | - | - |
| SHC 3 | *Bacillus circulans* strain MD1, KT757520.1 | 99 | 718 | MG051324 | | | | | | |
| DCB2-2 | *Paenibacillus polymyxa* strain DSM 36, NR_117733.2 | 99 | 643 | MG051296 | - | - | + | - | - | - |
| B2/38-2 | *Paenibacillus glucanolyticus* strain P_11, KT427632.1 | 99 | 909 | MG051306 | - | - | - | - | - | + |
| B2/38-3 | *Paenibacillus glucanolyticus* strain P_11, KT427632.1 | 99 | 898 | MG051307 | - | - | + | - | - | + |
| B2/38-4 | *Paenibacillus glucanolyticus* strain P_11, KT427632.1 | 99 | 921 | MG051308 | - | - | + | - | - | + |

# Supplementary Figures

**Supplementary Figure S1.**

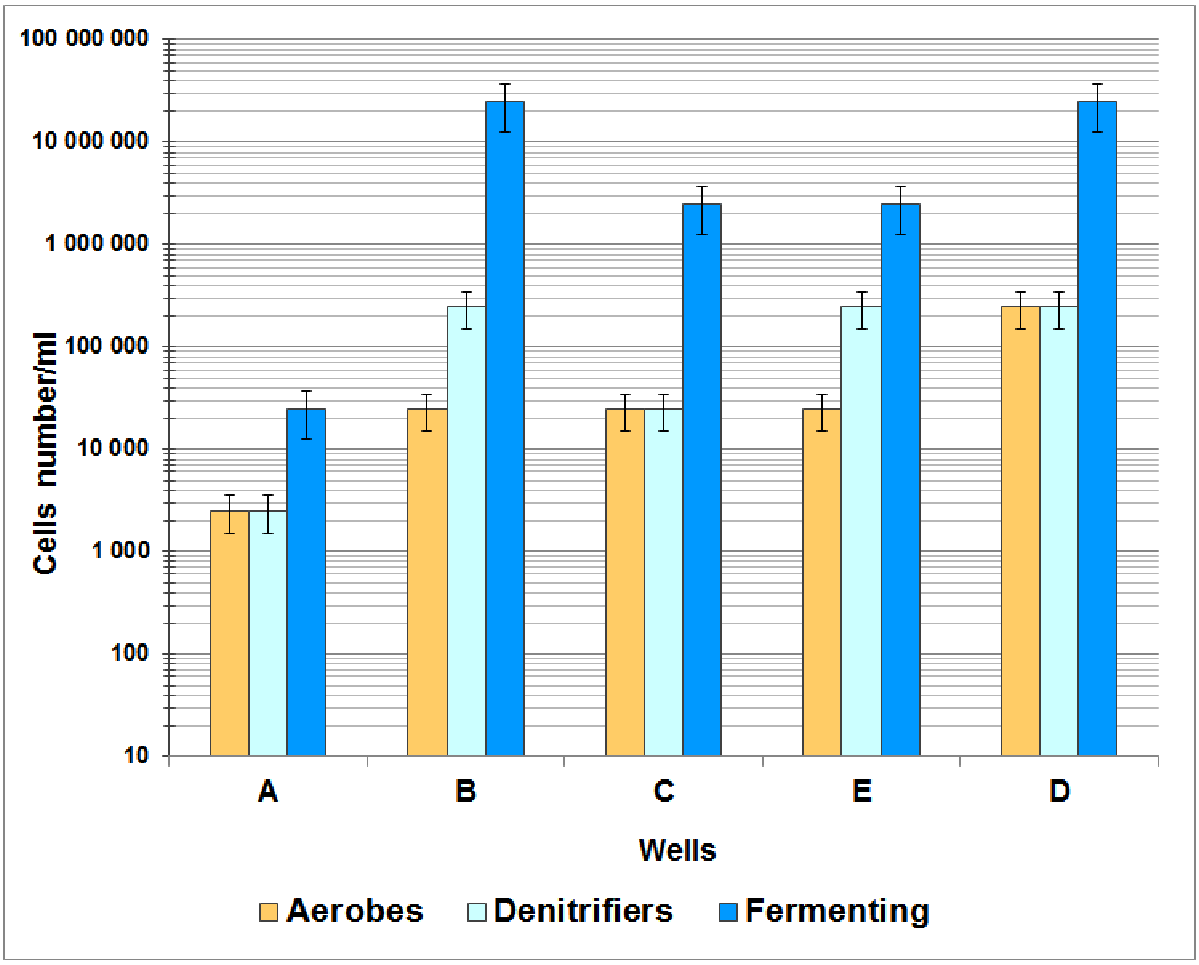


**Supplementary Figure S1 |** Numbers of aerobic and anaerobic microorganisms in groundwater samples at the area of the radioactive waste surface repository (July, 2017).

**Supplementary Figure S2**

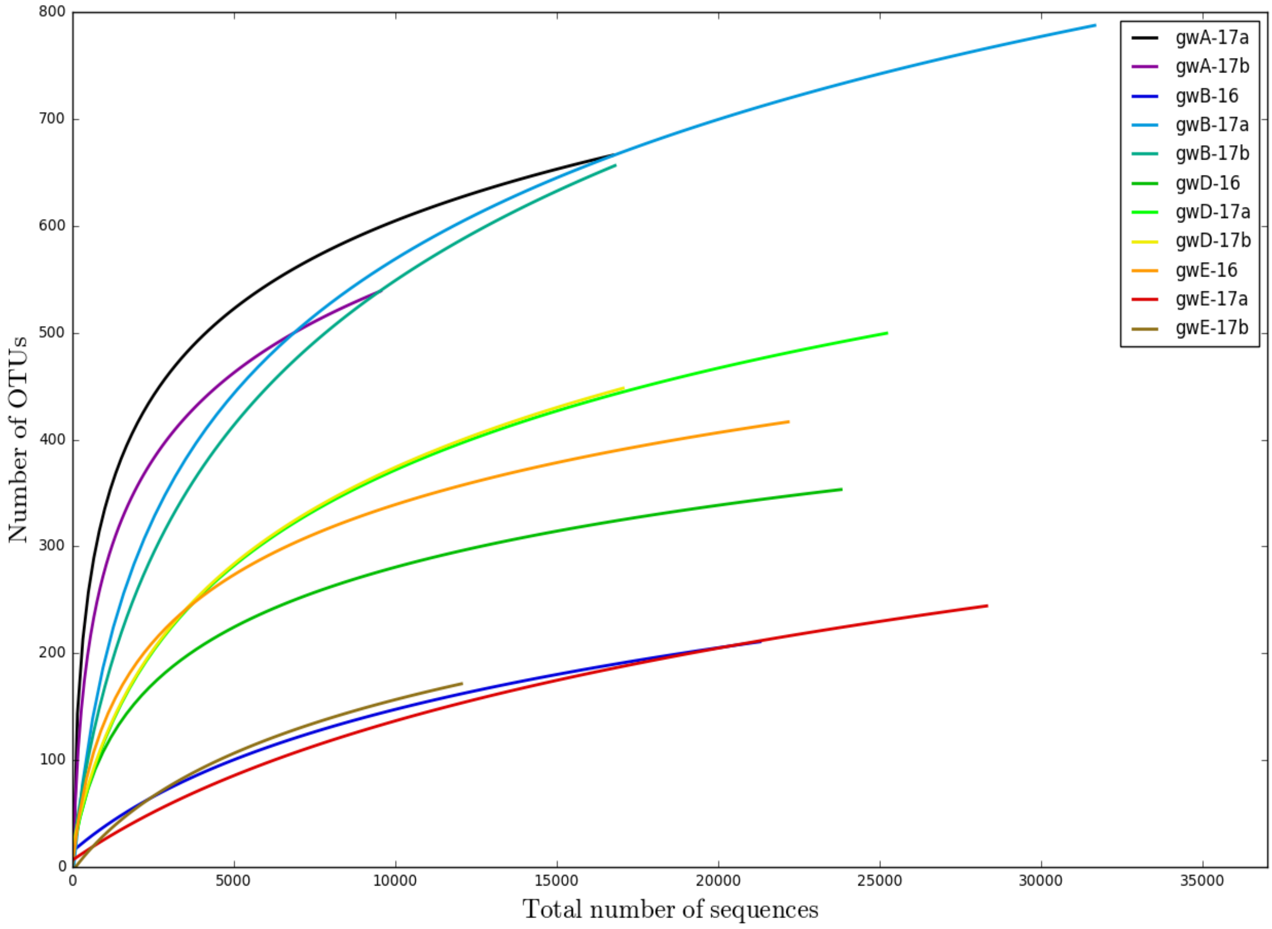


**Supplementary Figure S2 |** Rarefaction curves showing dependence of the number of OTUs of the 16S rRNA gene fragments from the total number of fragments in the library obtained from the DNA of groundwater samples. The sequences were grouped into phylotypes (operational taxonomic units) with similarity level ≥ 98%.

## Supplementary Figure S3|

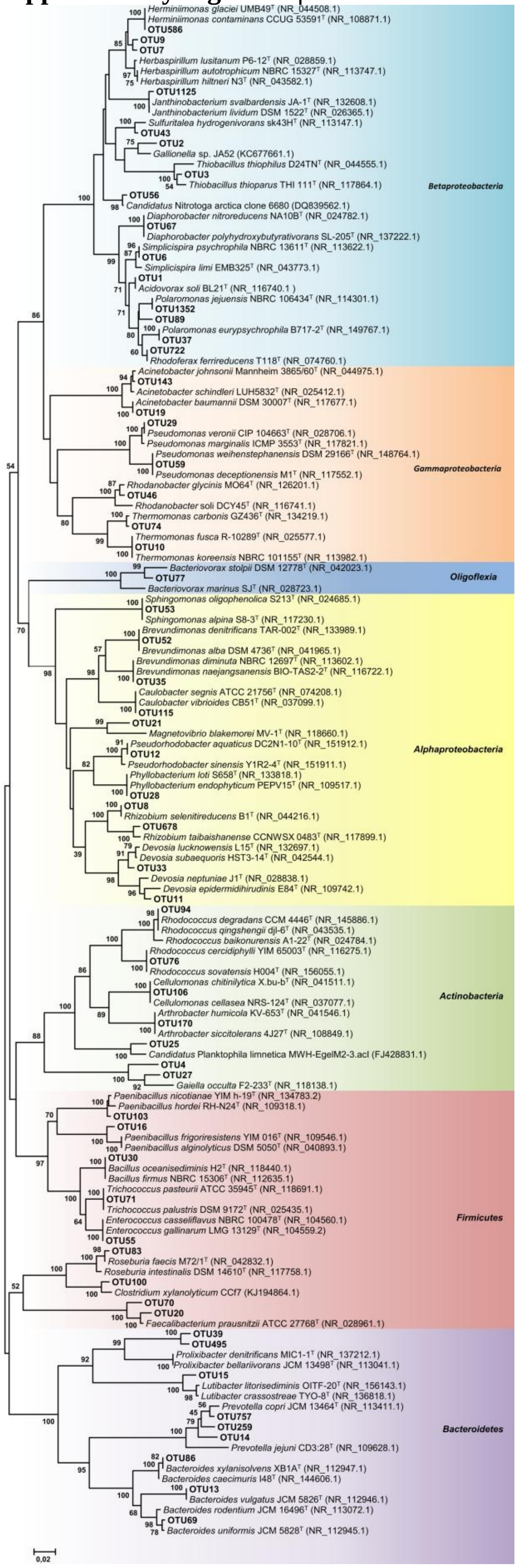

**Supplementary Figure S3 |** Phylogenetic tree showing the most abundant OTUs. Phylogeny is based on 400-bp alignment of the 16S rRNA gene. The tree was constructed using the neighbor-joining algorithm with the application of the Tamura–Nei model. The branching order was determined by bootstrap analysis of 1000 alternative trees.

**Supplementary Figure S4.**

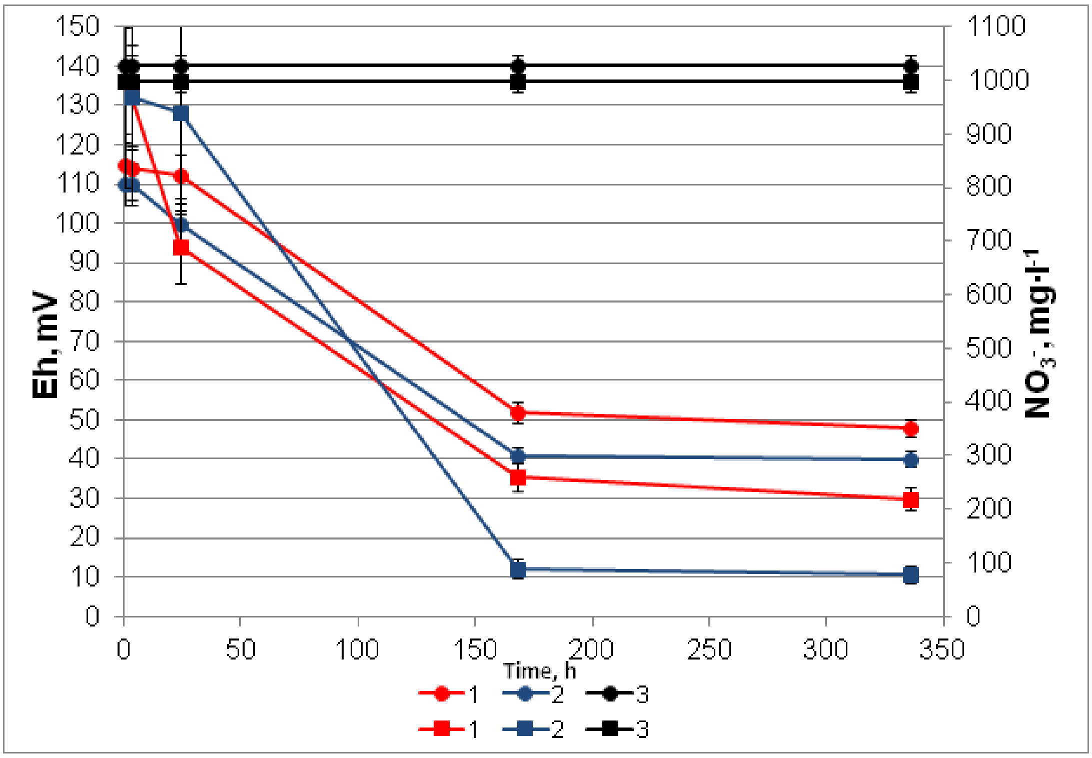


**Supplementary Figure S4 |** Redox potential (●) and nitrate concentration (■) in the medium during growth of denitrifying bacteria *P. veronii* SHC-8-1 (1) and *E. adhaerens* SHC-2-14 (2), and in sterile medium (3).

**Supplementary Figure S5**

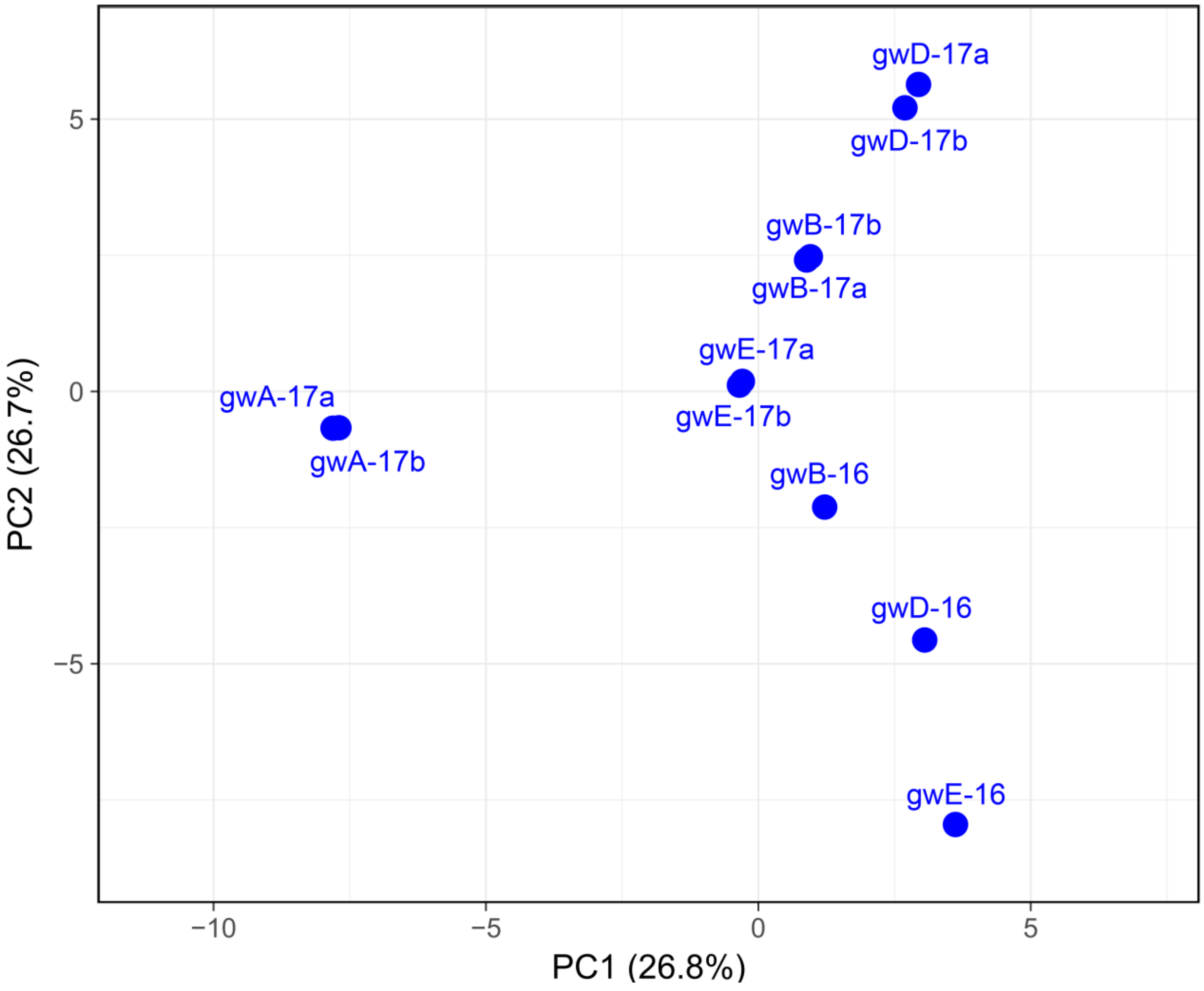


**Supplementary Figure S5 |** Principal component analysis (PCA) based on relative abundance of 16S rRNA gene OTUs from groundwater samples.
